# A fast, accurate and oscillation-free spectral collocation solver for high-dimensional transport problems

**DOI:** 10.1038/s41598-025-16905-6

**Published:** 2026-01-06

**Authors:** Nicola Cavallini, Gianmarco Manzini, Daniele Funaro, Andrea Favalli

**Affiliations:** 1 European Commission, Joint Research Centre, Via Enrico Fermi, 21027 Ispra, Italy; 2 Los Alamos National Laboratory, P.O. Box 1663, Los Alamos, NM 87545 USA; 3 Dipartimento di Scienze Chimiche e Geologiche, Università di Modena e Reggio Emilia, Via Campi 103, 41125 Modena, Italy; 4 Istituto di Matematica e Applicata e Tecnologie Informatiche del CNR, Via Ferrata 1, 27100 Pavia, Italy

**Keywords:** Engineering, Mathematics and computing, Physics

## Abstract

Transport phenomena—describing the movement of particles, energy, or other physical quantities—are fundamental in various scientific disciplines, including nuclear physics, plasma physics, astrophysics, engineering, and the natural sciences. However, solving the associated seven-dimensional transport equations poses a significant computational challenge due to the curse of dimensionality. We introduce the Tensor Train Superconsistent Spectral (${\hbox {T}}^2{\hbox {S}}^2$) solver to address this challenge, integrating Spectral Collocation for exponential convergence, Superconsistency for stabilization in transport-dominated regimes, and Tensor Train format for substantial data compression. ${\hbox {T}}^2{\hbox {S}}^2$ enforces a dimension-wise superconsistent condition compatible with tensor structures, achieving extremely low compression ratios, such as $\mathscr {O}(10^{-12})$, while preserving spectral accuracy. Numerical experiments on linear problems demonstrate that ${\hbox {T}}^2{\hbox {S}}^2$ can solve high-dimensional transport problems in minutes on standard hardware, making previously intractable problems computationally feasible. This advancement opens new avenues for efficiently and accurately modeling complex transport phenomena.

## Introduction

Transport phenomena are fundamental across a wide range of scientific disciplines, including nuclear physics, plasma physics, high-energy density physics, astrophysics, condensed matter physics, atmospheric science, oceanography, and engineering. These processes describe the movement and interaction of particles, energy, and other physical quantities within various media. At the heart of modeling these phenomena are transport equations, which comprehensively describe how quantities propagate and interact. For example, the neutron transport equation is fundamental in applications such as reactor design and radiation detection instrumentation, while the Vlasov equation is pivotal in plasma physics, describing the evolution of charged particles under electromagnetic fields, and is fundamental in applications ranging from fusion to space science.^1–6^ Although the precise nature of transport phenomena may vary across applications, the underlying mathematical structure remains consistent. A significant challenge in solving transport equations is the associated computational cost, given that a general solution spans seven dimensions: three spatial coordinates, two angles, one energy (or speed), and one time. As the number of dimensions increases, the required memory and computational resources scale exponentially, limiting the feasibility of traditional numerical methods. The challenge has been aptly described by F. Graziani and G. Olson as “Conquering the Seven-Dimensional Mountain”^7^.

To establish a robust computational framework, we focus on a fundamental yet representative model of transport phenomena: the linear, time-dependent, convection-diffusion-reaction PDE. This is given by:

1 $$\begin{aligned} \frac{\partial f}{\partial t} -\varepsilon \Delta _{\bf{x}}f+{\varvec \beta }\cdot \nabla _{\bf{x}}f+\rho f= b, \qquad \forall (\bf{x},t)\in \Omega \times ]0,T]\phantom {[[}, \end{aligned}$$

the equation describes the evolution of the distribution function $f(\bf{x},t)$ in the seven-dimensional phase space $\Omega \times [0,T]$, with $\bf{x} = (x_{1},x_{2},\ldots ,x_{d})^T\in \Omega \subset {\mathbb {R}}^{d}$, $d = 6$. Time is represented by $t\times [0,T]$ and $x_1, x_2, x_3$ refer to the 3-D spatial variables. Depending on the specific form of Eq. (1) $x_4, x_5, x_6$ either describe the momentum vector or $x_4, x_5$ represent the 2-D angular direction, and $x_6$ the energy or speed. The computational domain $\Omega \subset {\mathbb {R}}^{d}$ is the *d*-dimensional, open hypercube with characteristic edge length *L* and five-dimensional boundary $\Gamma$. To have a mathematically well-posed model, we assume that *f* satisfies Dirichlet boundary conditions on the domain boundary

2 $$\begin{aligned} f = g\quad \text {on}~\Gamma \times [0,T], \end{aligned}$$

and an initial condition at $t=0$

3 $$\begin{aligned} f(\cdot ,0)= f_0\quad \text {in}~\Omega . \end{aligned}$$

In Eq. (1), $\varepsilon$ is the diffusion coefficient, ${\varvec \beta }$ is the convection field, $\rho$ is the reaction field, and *b* is the right-hand side forcing term. As a matter of notation, in the latter we drop the $\bf{x}$ subscript in the differential operators notation: $\nabla _{\bf{x}}$ becomes $\nabla$ and $\Delta _{\bf{x}}$ becomes $\Delta$.

The numerical solution of high-dimensional problems in the framework of Partial Differential Equations (PDEs) faces a fundamental challenge often referred to as the *curse of dimensionality*^8^, where both memory requirements and computational costs scale exponentially with the number of dimensions. This is a serious limit for the effectiveness of numerical methods. While recent approaches have attempted to address this challenge through GPU acceleration^9^ and machine learning methods^10,11^, these solutions introduce further complexities, as they require either architecture-specific programming or extensive training data.

To address these computational challenges, we take a different path based on a three-level approach. First, we operate in the context of the *low-rank approximation*^12^. We adopt the Tensor Train format^13^, a linear algebra framework capable of efficiently handling high-dimensional data and mathematical operators, organized on tensor-product grids. Considerable effort has been devoted over the years to solving PDEs in Tensor Train format. Dolgov and coauthors tackled the three-dimensional Fokker-Planck equation^14^. Basic numerical schemes, such as uniform finite differences applied to the high-dimensional Poisson equation, are commonly used as testbed for the development of the Tensor Train linear system solvers^15,16^. A hybrid approach for parametric PDEs in two dimensions can be found in^17^. In the context of semi-Lagrangian solvers, where special care has to be devoted to conservation of mass, energy, or momentum, Kormann^18^ proposed a Tensor Train solver for Vlasov Equations.

Second, we numerically discretize the problem (1) using a variant of Spectral Collocation methods. The term “collocation” indicates that the continuous model, i.e. the PDE, is evaluated, “collocated”, at specific grid points. These techniques are known for their long-recognized exceptional *accuracy*^19,20^, commonly exhibiting an exponential rate of convergence for smooth solutions. This rate of convergence is also addressed as spectral accuracy. However, their application to higher-dimensional problems, in standard algebra format, has been constrained by the dense structure of the discrete operators and the complexity of solving the resulting linear systems^19,21^.

Third, these methods face a fundamental trade-off between *accuracy* and *stability*^22,23^. This becomes particularly problematic in regimes dominated by first-order derivatives ($\varepsilon /{|{\varvec \beta }L|}\ll 1$), where solutions present spurious oscillations that make the result unreliable. Funaro’s concept of superconsistency^24^ cures such oscillations by performing collocation on modified nodes. The new position of the nodes is a function of the ratio $\varepsilon /{|{\varvec \beta }L|}$ and the grid density^21,25,26^. We should note that superconsistency alone does not reduce the computational costs; the solution of high-dimensional problems remains prohibitive.

Our approach, *the Tensor Train Superconsistent Spectral* (${\hbox {T}}^2{\hbox {S}}^2$) method, builds on the three techniques described above. It addresses the limitations of each individual block through a novel dimension-wise application of the superconsistent method. This approach fundamentally redefines the original superconsistent framework, making it compatible with the Tensor Train format and enabling an efficient storage of the mathematical operators. The method preserves the hallmark exponential accuracy of Spectral Collocation methods, while maintaining the stabilization properties with respect to the $\varepsilon /{|{\varvec \beta }L|}$ ratio. At the same time ${\hbox {T}}^2{\hbox {S}}^2$ achieves extremely low compression ratios, such as $\mathscr {O}(10^{-12})$. As a result, it can solve high-dimensional transport problems in minutes on standard hardware, rendering previously intractable problems computationally feasible.

The paper is organized as follows. The first section, “Overview of the ${\hbox {T}}^2{\hbox {S}}^2$ Approach”, presents the method, followed by the second section, “Results”, which demonstrates the validation of the method through numerical experiments. The third section, “Discussion”, summarizes the method’s strengths, applications and future directions. The fourth section, “Methods”, provides the detailed mathematical framework underlying the ${\hbox {T}}^2{\hbox {S}}^2$ solver.

## Overview of the ${\hbox {T}}^2{\hbox {S}}^2$ approach

**Fig. 1:**
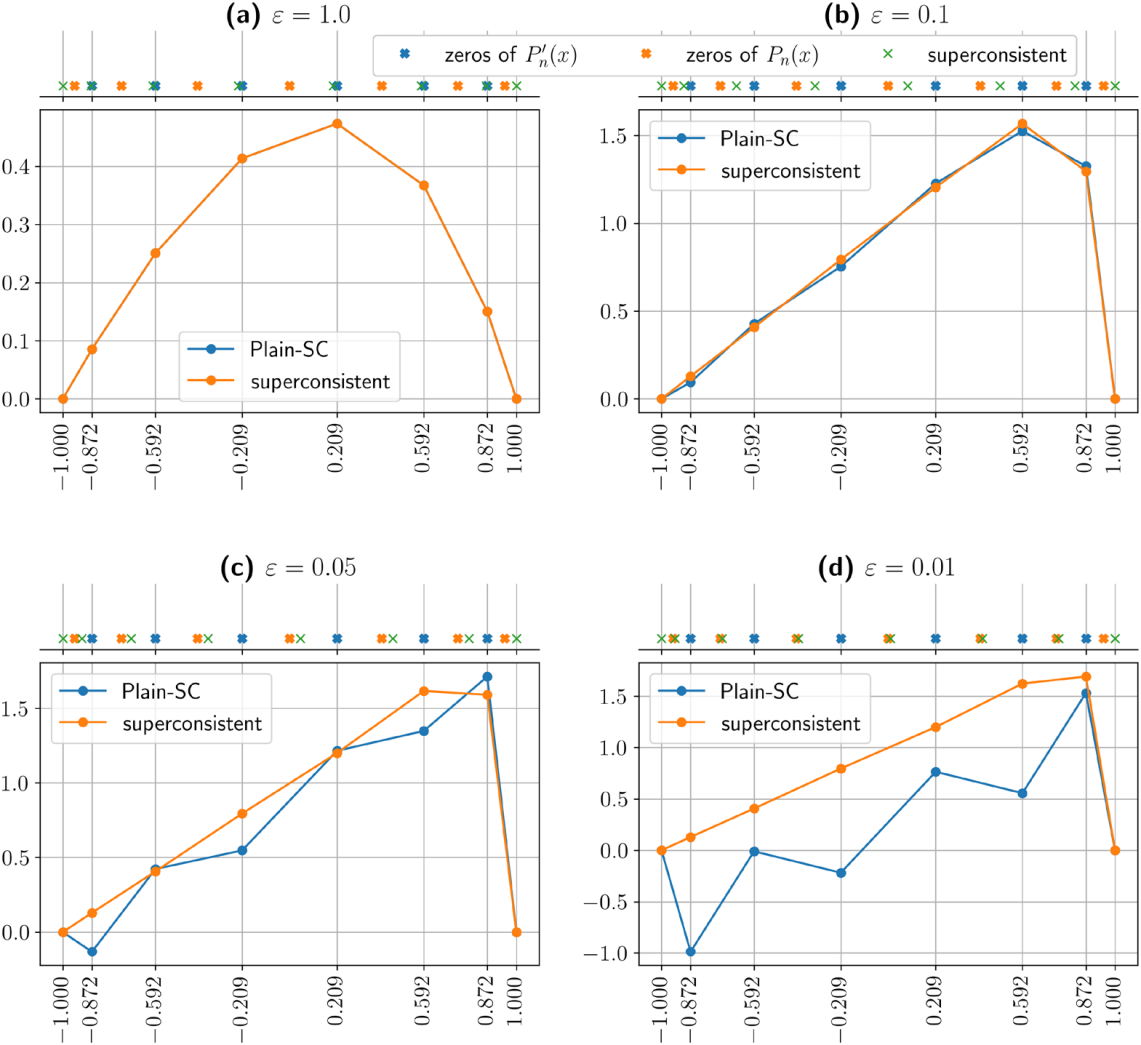
Superconsistent approximation of Eq.(4) for the polynomial degree $n=7$, and $\beta =1$. On the top horizontal axis, the blue crosses mark the zeros of $P'_n$, optimal for diffusion; the orange crosses mark the zeros of $P_n$, optimal for convection; the green crosses mark the superconsistent collocation points for mixed regimes. **(a,b)** Relatively accurate solutions when convection and diffusion are balanced, in particular in panel **(a)** the two solutions almost coincide. As convection dominates **(c,d)**, the Plain-SC solution is unreliable, whereas the superconsistent one does not present problems.

This section outlines the key components of our methodology and demonstrates how they synergize to create our computational framework. We illustrate the underlying principles through one- and two-dimensional examples, while the detailed mathematical framework is provided in section “Methods”.

(i) **Spectral collocation methods.** Spectral collocation relies on two fundamental elements: *basis functions* and *representation points*. We employ Lagrange basis functions defined on $n+1$ representation points^21^, which are the $n-1$ zeros of $P_{n}^{\prime }$, the derivative of the *n*-th degree Legendre polynomial $P_n$, complemented with the two extrema of the interval $[-1, 1]$. We will refer to this approach as *plain spectral collocation* (Plain-SC) method. The solution to a one-dimensional transport problem is a linear combination of these basis functions. The coefficients are determined by solving the linear system obtained by *collocating the equation* at the same representation points, i.e., by evaluating the left-hand side and the right-hand side of Eq. (1) at such locations. For nomenclature clarity, in one dimension, the number of degrees of freedom ($\#\textsf{dofs}_{\textrm{1d}}$) equals $n+1$. The multidimensional extension of the method is achieved through the tensor product of the one-dimensional discretizations along all the problem’s directions, $\#\textsf{dofs}$ addresses the total number of degrees of freedom.

(ii) **Superconsistent formulation.** To illustrate the concept of superconsistency we consider the time-independent one-dimensional model

4 $$\begin{aligned} \beta f^{\prime}(x) - \varepsilon f^{\prime\prime}(x) = 1 \quad x\in\big [-1,1\big ], \quad f(-1) = f(1) = 0, \end{aligned}$$

where $\beta >0$ and $\varepsilon >0$ are the scalar convective and diffusion coefficients, respectively. The Plain-SC method, employing the zeros of $P_{n}^{\prime }$, (blue dots on the top axis of Fig. 1), develops spurious oscillations when the convective term becomes dominant relatively to the diffusive one, meaning that $\varepsilon /{|{\varvec \beta }L|}\ll 1$. This effect is clearly visible in panels (c) and (d) of Fig. 1.

Funaro^24^ cured this phenomenon by introducing the superconsistent condition. The superconsistent condition takes into account the differential operator and is satisfied by introducing a different set of *collocation points* where the partial differential equation and the right-hand side are evaluated.

According to the superconsistent condition, the zeros of $P_{n}'$ are optimal for the approximation of the diffusion operator $\varepsilon f^{\prime }$, while the zeros of $P_{n}$ are optimal for $\beta f^{\prime }$. In hybrid regimes, the superconsistent points establish an optimal intermediate distribution between these two configurations. As illustrated in Fig. 1, when convection and diffusion terms are balanced (Fig. 1a), the superconsistent points are close to the zeros of $P'_n$. As the diffusion coefficient $\varepsilon$ decreases maintaining *n* fixed (Fig. 1b, c), the superconsistent points shift towards the zeros of $P_{n}$. In the convection-dominated regime (Fig. 1d), the superconsistent points nearly coincide with the zeros of $P_{n}$.

In summary, the superconsistent approach is based on two grids: the *representation grid* and the actual *collocation grid*. The position of the collocation grid depends on the ratio $\varepsilon /{|{\varvec \beta }L|}$ and *n*. Enforcing the superconsistent condition provides the numerical strategy to locate the collocation points in order to achieve stabilization with respect to $\varepsilon /{|{\varvec \beta }L|}$. We use the representation points to build the Lagrange basis functions. These basis functions are used to evaluate the discrete operators at the collocation grid. The result of this *collocation* operation, together with boundary conditions, leads to the construction of the final linear system.

(iii) **Tensor-train format.** Traditional spectral methods struggle with the *curse of dimensionality*^8^, where computational complexity increases by scaling exponentially with the number of dimensions.

**Fig. 2:**
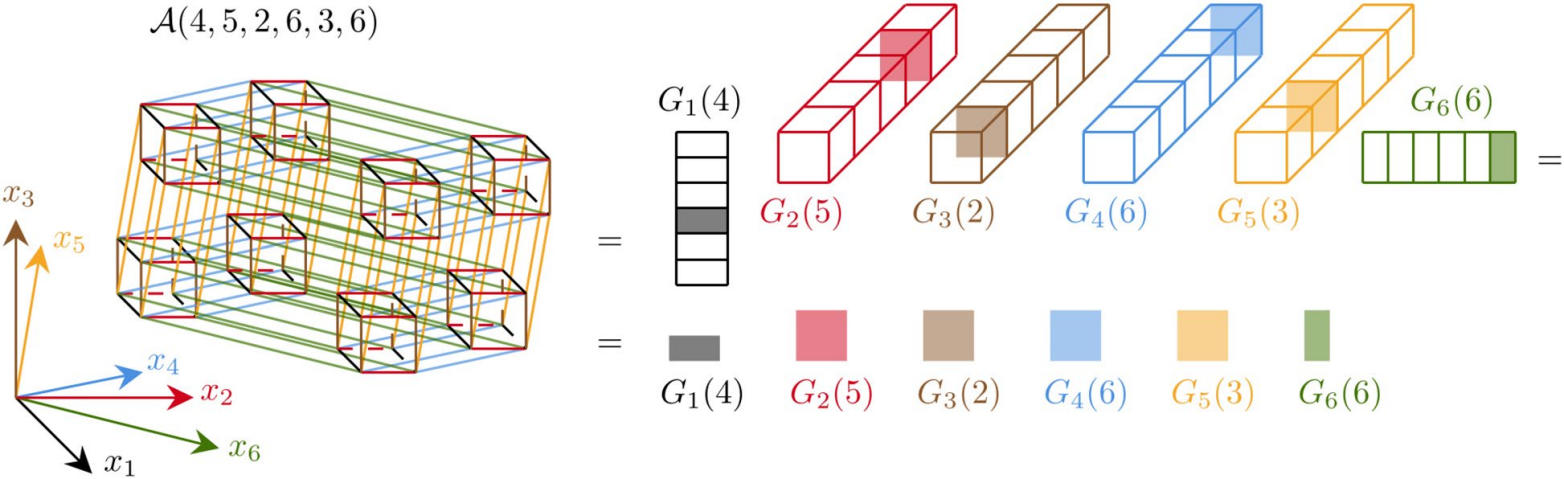
Focusing on the six spatial dimensions, a six-dimensional array $\mathcalligra{\scriptstyle A}\:$ has size $n^6$, with $x_1, x_2, \ldots , x_6$ coordinate axis of the six-dimensional space. The TT format represents the whole array on the left as the product of six cores on the right. The $\ell$-th core for $\ell =1,2,\ldots ,6$ has size $r_{\ell -1}\times n\times r_{\ell }$, with $r_{0}=r_{6}=1$, so the first and last cores are matrices.  The (4, 5, 2, 6, 3, 8)-th entry of the full tensor is represented in the TT-format as the product $\mathcalligra{\scriptstyle A}\:(4,5,2,6,3,8)=G_{1}(4)G_{2}(5)G_{3}(2)G_{4}(6)G_{5}(3)G_{6}(8)$, i.e., as the product of the 4-th, 5-th, 2-nd, 6-th, 3-rd, 8-th slices of the corresponding cores.

The Tensor-train (TT) format^13^ efficiently represents a $d$-dimensional tensor as the product of $d$ three-dimensional arrays, the *TT cores*, significantly reducing the memory requirement. TT core, denoted by $G_{\ell }$ and indexed by $\ell =1,2\ldots ,d$, has dimensions $r_{\ell -1}\times n_{\ell }\times r_{\ell }$, where $n_{\ell }$ is the original dimension size also called the *mode size*, and $r_{\ell }$ are the *TT-ranks* (with $r_{0}=r_{d}=1$). As shown in Fig. 2 for a six-dimensional tensor, an element of the multidimensional array $\mathcalligra{\scriptstyle A}\in \mathbbm {R}^{n_{1}\times \ldots n_{6}}$ is reconstructed by multiplying the corresponding core slices $G_{\ell }\in \mathbbm {R}^{r_{\ell -1}\times n_{\ell }\times r_{\ell }}$, $\ell =1,2,\ldots ,6$. The TT decomposition is not unique and is most effective when the TT-ranks are much smaller than the mode size, i.e., for $r_{\ell }\ll n_{\ell }$. In such a case, this method efficiently compresses large multidimensional arrays and, when applied to operators, mitigates the high bandwidth issue arising from tensor products.

We build the ${\hbox {T}}^2{\hbox {S}}^2$ solver combining principles (*i*), (*ii*), and (*iii*) to extend superconsistency to high-dimensional equations. Our approach implements the superconsistent condition independently along each dimension, treating them as decoupled problems. This formulation works with two distinct time integration frameworks: the *unified space-time formulation*^27^ that handles time as an additional dimension, and the *method-of-lines formulation*^28,29^ that uses conventional time-stepping schemes. For the latter, we implement the Crank–Nicolson and the backward Euler schemes. Highly accurate variants of these schemes, such as^30^ are combinations of the mentioned methods. Superconsistency is applied only to the six spatial dimensions, demonstrating an oscillatory-free performance with respect to the ratio $\varepsilon /{|{\varvec \beta }L|}$.

**Fig. 3:**
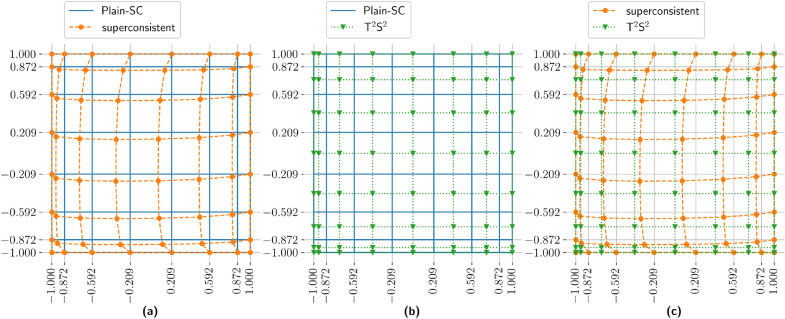
Left panel: Plain-SC nodes and Funaro’s superconsistent nodes^21^. Such a grid has been evaluated for problem (4) with parameters $\varepsilon ={1}/{50}$, $\beta =(1,0.5)^T$ and $n=7$. Middle panel: Plain-SC nodes and ${\hbox {T}}^2{\hbox {S}}^2$ collocation nodes, which have been independently computed in each direction. Right panel: Comparison between the 2D Funaro’s superconsistent nodes^21^ and ${\hbox {T}}^2{\hbox {S}}^2$ collocation nodes.

In Fig. 3 we show a two dimensional grid obtained using ${\hbox {T}}^2{\hbox {S}}^2$. The resulting Cartesian structure naturally fits with the TT format and extends straightforwardly to higher dimensions. This approach differs from Funaro’s original formulation^21^ , which applies superconsistency to the full multidimensional problem and produces non-Cartesian grid deformations, see Fig. 3. Our method is stable across physically relevant parameter ranges and preserves the exponential accuracy that is characteristic of the spectral collocation methods.

## Results

In this section, we analyze the ${\hbox {T}}^2{\hbox {S}}^2$ performance from various perspectives. First, we assess its accuracy and convergence by investigating its behavior when approximating a low-rank, high-frequency analytical solution. The high-frequency test examines ${\hbox {T}}^2{\hbox {S}}^2$’s performance on a grid density with $300^7\approx 2.2\, 10^{17}$ nodes that can only be explored by low-rank solvers since a full format traditional solver, even at minimal complexity, would require millions of years on modern supercomputers. Next, we present a six-dimensional test case that triggers the $\varepsilon /{|{\varvec \beta }L|}\ll 1$ instabilities when we approximate a high-gradient solution that challenges the low-rank approximation on moderate to high-density grids. It tests the robustness of ${\hbox {T}}^2{\hbox {S}}^2$ in handling such instabilities. Finally, we complete our investigation with two low-dimensional test cases that challenge the non-oscillatory performance of the ${\hbox {T}}^2{\hbox {S}}^2$ solver: the “traveling bump” benchmark^21,31^ and the Hughes double-layer benchmark^32,33^.

### Global accuracy and computational cost

Testing numerical algorithms with manufactured analytical solutions is a cornerstone practice in numerical analysis. It provides a reliable way to create benchmark solutions for validating numerical solver^34^. In this case, we construct an “exact” solution by choosing a suitable expression, and we determine the corresponding right-hand side analytically. To this end, we apply our ${\hbox {T}}^2{\hbox {S}}^2$ solver to problem (1)–(3) on $\Omega \times [0,T]$ with $\Omega =[-1,1]^6$ and $T=1$. For convenience, we rescale the time interval to match the interval $[-1,1]$. We set $\varepsilon =10^{-4}$, and define:

$$\begin{aligned} {\varvec \beta }(x_1,x_2,\ldots ,x_6)&= ( \sin (\pi x_{1}), \sin (\pi x_{2}), \ldots , \sin (\pi x_{6}) )^T,\\ \rho (x_1,x_2,\ldots ,x_6)&= \prod _{1\le i\le 6}\left[ \frac{1}{2}-\left( x_i+\frac{11}{10}\right) \left( x_i-\frac{1}{2}\right) \left( x_i+\frac{1}{2}\right) \left( x_i-\frac{11}{10}\right) \right] . \end{aligned}$$

The right-hand side *b* and the Dirichlet boundary conditions are determined from the proposed analytical solution:

$$\begin{aligned} f({\bf x},t) = e^{t} \cdot \prod _{1\le i\le 6}\left( \sin (\pi x_{i}) + 0.3\sin (80\pi x_{i}) \right) . \end{aligned}$$

Here, we test the space-time formulation. In this setting, the initial condition is naturally incorporated as a boundary condition at $t = -1$, while at $t = 1$, the numerical solution is a result of the numerical scheme. The analytical solution $f({\bf x},t)$ incorporates two spatial components: a low-frequency one, i.e. $\sin (\pi \cdot )$, that spans the computational domain, and a high-frequency one, i.e. $\sin (80\pi \cdot )$, that introduces features at approximately 1/100 of the domain size. Such multi-scale phenomena are commonly encountered in practical applications^35,36^.

**Fig. 4:**
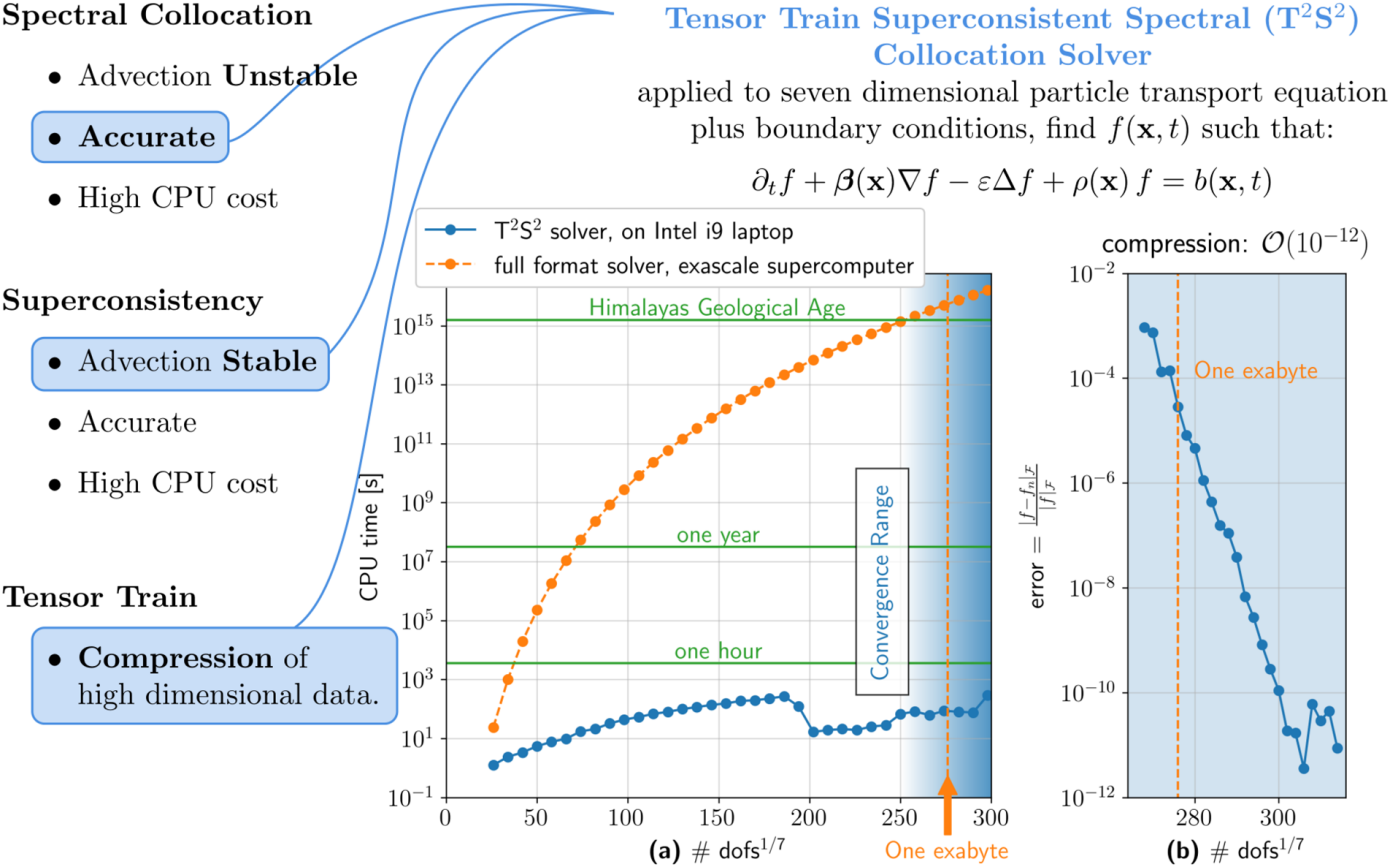
This figure showcases the key components of ${\hbox {T}}^2{\hbox {S}}^2$ and its performance in solving the manufactured transport problem. (**a**) Illustrates ${\hbox {T}}^2{\hbox {S}}^2$’s compression capabilities, which enable the real-time solution of a seven-dimensional space-plus-time problem. To convey to the reader the scale of the performance, we provide a comparison with the estimated computational time for a full format solver with minimal numerical complexity^19,21^, whose cost scales as $\mathcalligra{\scriptstyle O}\:((\#\textsf{dofs}_{\textrm{1d}})^7)^2$. Assuming such a full format solver runs on a 2.7 exaFLOPS nominal supercomputer, the projected execution time would be on the order of fifty million years, comparable to the geological age of the Himalayas. An arrow is used to indicate the “One exabyte” memory for the full format solver. (**b**) The convergence behavior. The error decreases exponentially to $\mathcalligra{\scriptstyle O}\:(10^{-11})$.

The results are presented in Fig. 4. Each “dot” represents an approximation of the solution of problem (1) for a given polynomial degree (associated with the number of degrees of freedom). The convergence tests are based on the following $L^2$-type error norm:

$$\begin{aligned} \textrm{error} = \frac{|\hspace{-0.2mm}|f-f_{n}|\hspace{-0.2mm}|_{\mathcalligra{\scriptstyle F}}}{|\hspace{-0.2mm}|f|\hspace{-0.2mm}|_{\mathcalligra{\scriptstyle F}}}\:, \end{aligned}$$

where $|\hspace{-0.2mm}|,\cdot ,|\hspace{-0.2mm}|_{\mathcalligra{\scriptstyle F}}\:$ denotes the Frobenius norm and $f_{n}$ represents the polynomial approximation of degree $n$ of the solution $f$.

Panel ${(b)}$ shows that we need a grid of approximately $256^7$ size to capture the two dominant frequencies of the solution. From that point onwards, we can appreciate the exponential decay of the error characteristic of the spectral methods. The solver achieves an $\mathcalligra{\scriptstyle O}\:(10^{-11})$ approximation error for a grid size having a total number of $300^7$ nodes (including time). Panel ${(a)}$ shows that ${\hbox {T}}^2{\hbox {S}}^2$ achieves the desired accuracy in under three minutes on an Intel i9 laptop computer with 64 GBytes RAM. We put this number into perspective theorizing a full format Super Spectral Collocation solver with minimal quadratic complexity $((\#\textsf{dofs}_{\textrm{1d}})^7)^2$, labeled *full format solver*^21,24^. We estimate that a modern supercomputer, characterized by a peak performance of $2.7^{18}$ floating point operations per second^37^, would require approximately fifty million years to run the full format solver on the $256^7$ grid. The computational cost for the first grid that exhibits spectral accuracy, the one with $300^7$ total degrees of freedom, would approximately be 300 million years. These two timescales respectively compare with the geological age of the Himalayas and the Dolomites. These results are a consequence of the fact that ${\hbox {T}}^2{\hbox {S}}^2$ can capture a low-rank solution of a maximum rank of 9, resulting in a compression ratio of $(7\cdot 9^{2}\cdot 300 ) /{300^7}=\mathcalligra{\scriptstyle O}\:(10^{-12})$. The corresponding memory requirement for the full format solver exceeds one exabyte, indicated by the arrow in Fig. 4${(a)}$. One exabyte compares at best, or exceeds, the storage capacity of todays largest supercomputers^37^. The small drop in the computational time for ${\hbox {T}}^2{\hbox {S}}^2$ depends on the fact that the solver, on the finer meshes, is closed to the real solution and in general needs less iterations, or swaps in the TT nomenclature, to get to convergence.

**Fig. 5:**
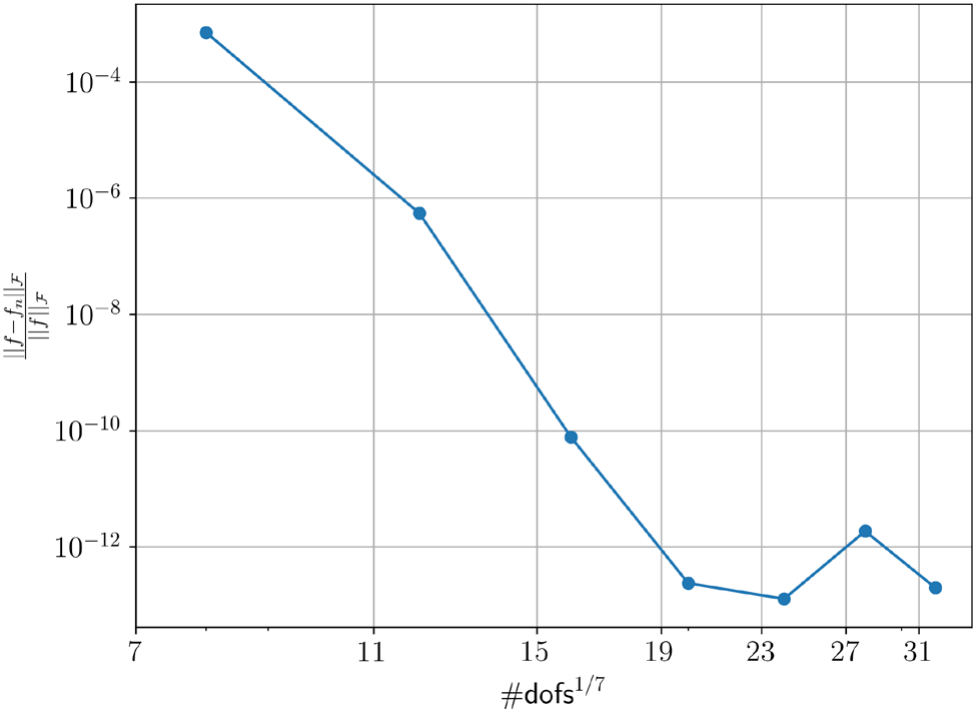
This convergence curve shows the exponential decay of the approximation error of the numerical solution versus the number of degrees of freedom in each direction from the application of ${\hbox {T}}^2{\hbox {S}}^2$ to a seven-dimensional convection-diffusion problem with velocity field ${\varvec \beta }=(1,1,\ldots ,1)^T$ and diffusion coefficient $\varepsilon =10^{-6}$.

Figure 5 shows the convergence analysis of ${\hbox {T}}^2{\hbox {S}}^2$ on a six-dimensional, constant coefficient test case. We set $\varepsilon =10^{-6}$, ${\varvec \beta }$ as a six-dimensional unit vector and $\rho =0$. The analytical solution is prescribed as

$$\begin{aligned} f({\bf x},t) = e^{-t} \cdot \prod _{ 1 \le i \le 6}\left( \sin (\pi x_i) \right) , \end{aligned}$$

from this, we derive the corresponding right-hand side and boundary condition for the discrete model. Figure 5 presents the error curve as a function of the number of degrees of freedom along one dimension. The results demonstrate that the method preserves spectral accuracy.

### Superconsistent stabilization of convection-dominated problems

Stabilization techniques for convection-diffusion problems have been extensively studied in the literature^32,33^, mainly focusing on low dimensional stationary convection-diffusion equation with Dirichlet boundary conditions. We consider a six-dimensional equivalent:

5 $$\begin{aligned} \begin{aligned} -\varepsilon \Delta f+ {\varvec \beta }\cdot \nabla f&= b\phantom {g}\quad \text {in}~\Omega ,\\ f&= 0 \phantom {b}\quad \text {on}~\Gamma . \end{aligned} \end{aligned}$$

This equation is a specialized case of our target problem (1), which we analyze on the six-dimensional hypercube domain $\Omega =[-1,1]^6$. Conventional spectral approximations perform effectively in diffusion-dominated regimes where $\varepsilon \Delta f$ predominates over ${\varvec \beta }\cdot \nabla$. However, they exhibit spurious oscillations in convection-dominated scenarios which are common in many practical applications^35,38,39^.

**Fig. 6:**
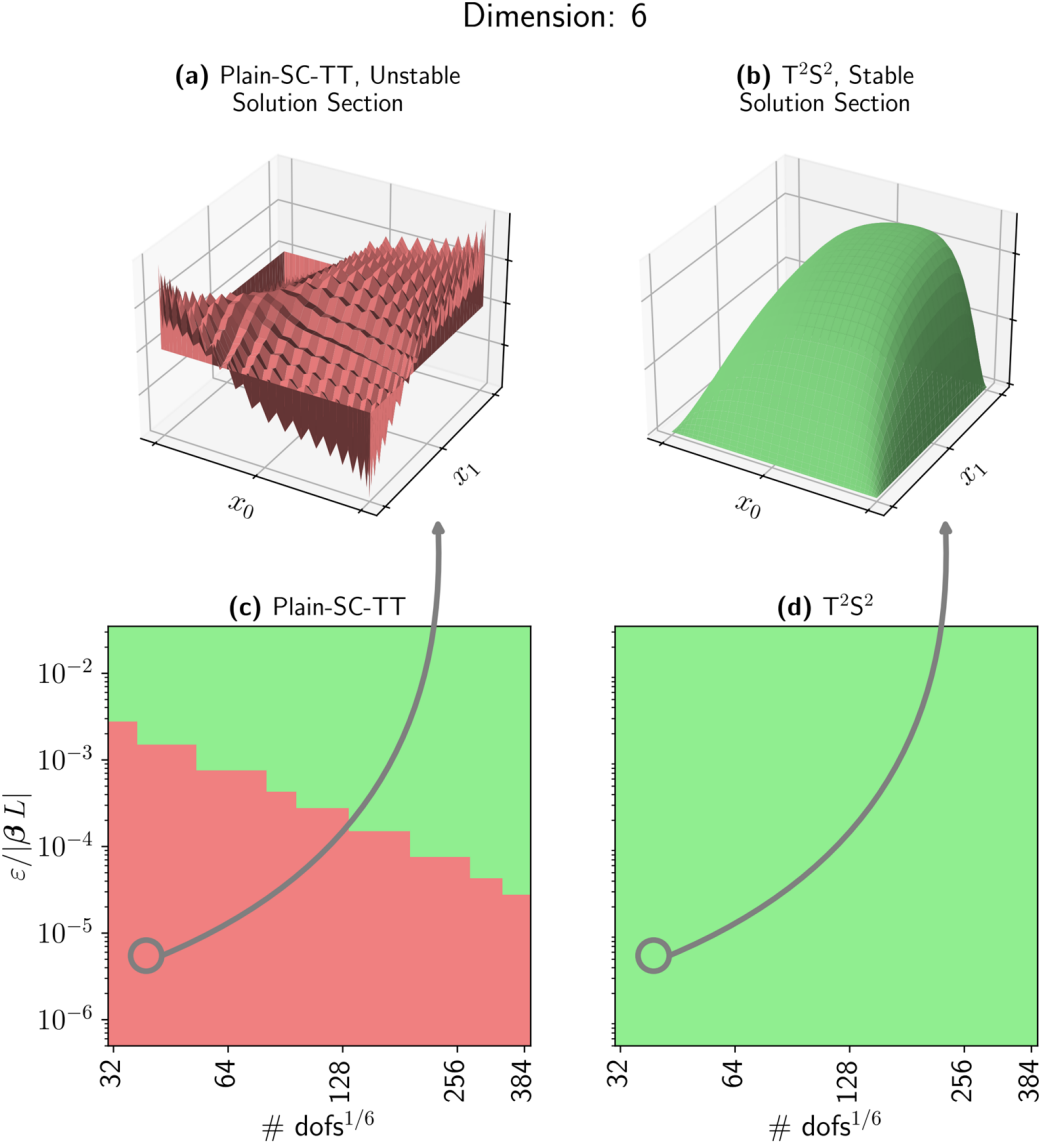
With ${\varvec \beta }$ fixed as a six-dimensional unit vector, we conduct a detailed analysis of the solution’s oscillatory behavior. (**a**) shows a two-dimensional cross-section of the solution to the six-dimensional problem (5) with $\varepsilon = 10^{-5}$ and polynomial degree 39, using collocation points at the zeros of $P'_n$. The solution presents macroscopic spurious oscillations. The corresponding solution using superconsistent collocation points exhibits the expected regularity and characteristic boundary layers. (**c,d**) Colormaps where each pixel represents a discrete solution of problem (5) for specific combinations of polynomial degree and $\varepsilon /{|{\varvec \beta }L|}$. (**c**) Highlights regions of oscillatory solutions in red, demonstrating the limitations of Plain-SC-TT. In contrast, (d) illustrates how our method maintains non-oscillatory behavior across all values of $\varepsilon /{|{\varvec \beta }L|}$ and polynomial degrees.

For our numerical experiments, we take $b=1$ and define ${\varvec \beta }$ as a vector of ones, and homogeneous boundary conditions are applied. In Fig. 6, we vary the degree of the space discretizations and $\varepsilon$. We compare ${\hbox {T}}^2{\hbox {S}}^2$ with the Plain-SC stored in TT format, we label this method Plain-SC-TT. The results are displayed on colormaps (c) and (d), where red pixels indicate an oscillatory solution; green pixels represent a non-oscillatory solution. We count the number of sign changes in the first derivative of the numerical solution. We compare this number with the expected sign changes in the regular solution. If the first count exceeds the second, the solution is marked as oscillatory. Oscillations are evaluated on the mid-axis along the first direction, at the central degree of freedom.

Panels ${(a)}$, ${(b)}$ of Fig. 6 show a two-dimensional slice of the six-dimensional solution computed with $\varepsilon =10^{-5}$ and 40 degrees of freedom. Panel ${(b)}$ presents the non-oscillatory solution, which exhibits a continuous profile with a well-resolved boundary layer at the domain perimeter. In contrast, panel ${(a)}$ shows the oscillatory solution, where spurious oscillations dominate the numerical approximation, rendering the spatial discretization ineffective. Colormap in Fig. 6(c) shows that the Plain-SC-TT approach is unstable for a wide range of parameters. The stable/unstable interface follows a precise law; diffusive terms scale like $\mathcalligra{\scriptstyle O}(\#\textsf{dofs}_{\textrm{1d}}^{4})$, while transport terms scale like $\mathcalligra{\scriptstyle O}(\#\textsf{dofs}_{\textrm{1d}}^{2})$, see section “Methods”.

It is, in principle, true that even for a very small value of $\varepsilon /{|{\varvec \beta }L|}$, we can find a sufficiently refined mesh where the diffusion term becomes numerically dominant, and the Plain-SC-TT approximation is stable. This approach is unfortunately illusive, since it is always possible to choose smaller values of $\varepsilon /{|{\varvec \beta }L|}$ that result in spurious oscillations in the solution. Moreover, the computational capability of TT linear solvers is remarkable but not infinite. AMEn^15^, an advanced TT linear system solver requires solving local problems at each TT core iteration, with each local direct solution having complexity $\mathcalligra{\scriptstyle O}((r_{\ell -1}, n_{\ell },r_{\ell })^{3})$, where $r_{\ell -1}, r_{\ell }$ denote the TT ranks and $n_{\ell }$ the mode size of the corresponding core. This results in a challenging computational limit on the maximum possible resolution determined by the hardware’s RAM. This is why ${\hbox {T}}^2{\hbox {S}}^2$ comes into play. The super-consistent discretization is always unconditionally stable regardless of the choice of the parameters; see Fig. 6(d).

The boundary layer in Fig. 6${(b)}$, characterized by a high gradient connecting the domain solution to the zero boundary condition challenges the rank compression of the TT format, as higher gradients typically result in a more dense solution structure in terms of dominant patterns^40^.

**Fig. 7:**
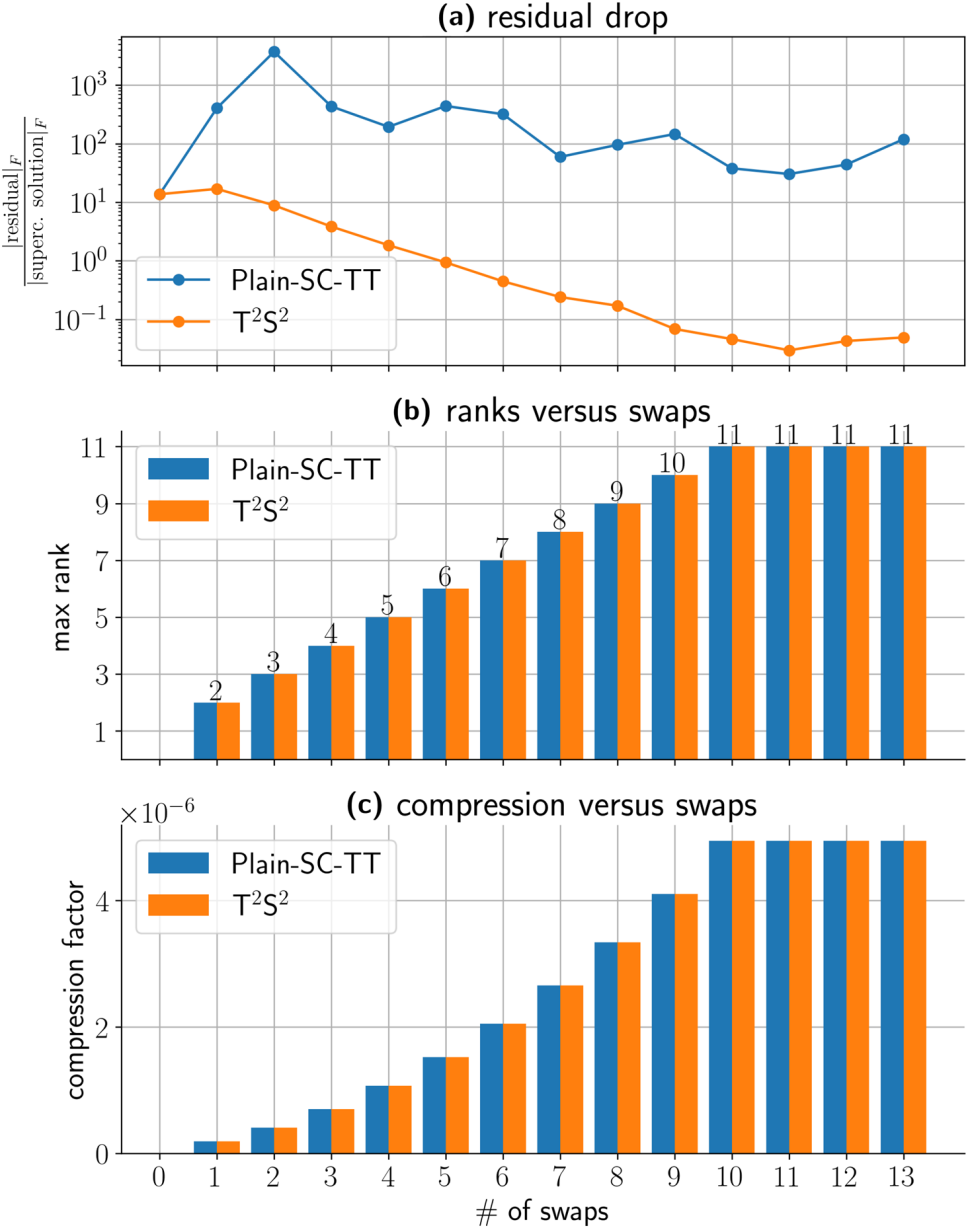
We present the residual decay, the ranks, and the compression factor against the number of AMEn swaps^15^ for our ${\hbox {T}}^2{\hbox {S}}^2$ solver and the Plain-SC-TT collocation methods in the case of 6 dimensional problem 5, degree 39, $\varepsilon =10^{-5}$ and ${\varvec \beta }$ six-dimensional vector of ones. This problem is of special interest because it presents a boundary layer. The boundary layer is a high-gradient region of the solution that connects a boundary condition with the internal values. The approximation of such high gradients is generally challenging in the TT format. (**a**) Reports how the residual drops against the number of swaps. The residual does not converge in the Plain-SC-TT case, while in ${\hbox {T}}^2{\hbox {S}}^2$ case, the residual drops with a regular gradient. The final value of the residual $\mathcalligra{\scriptstyle O}\:(10^{-2})$ is a consequence of the overall bad conditioning of the problem. (**b**) The solver increases the rank of the solution at each swap until it finds a stationary point at swap 11 for a corresponding rank equal to 11. After the 11th swap, the solver does not find any further information. The compression factor is in the range of $\mathcalligra{\scriptstyle O}\:(10^{-6})$. (**c**) The computational cost on an i9 Intel laptop with 64 Gigabytes of RAM is in the range of seconds.

In Fig. 7, we relate the linear solver “iterations” to the TT ranks, compression factors, and residuals for ${\hbox {T}}^2{\hbox {S}}^2$ and Plain-SC-TT. The TT solver is AMEn^15^, and the “iterations” are addressed as swaps in the TT nomenclature. Panel ${(b)}$ illustrates that the TT solver adjusts the solution rank with each swap, reaching a stationary point at swap 11 with a rank of 11. At this point, both our method and the Plain-SC-TT approach reach their resolution limit, and beyond it, they are unable to extract further information. Maximum rank 11 corresponds to a compression factor around $\mathcalligra{\scriptstyle O}(10^{-6})$, cf. the details in panels ${(b)}$ and ${(c)}$. Although ${\hbox {T}}^2{\hbox {S}}^2$ and Plain-SC-TT achieve a similar compression ratios, their convergence properties diverge substantially. The residual analysis in panel ${(c)}$ reveals that ${\hbox {T}}^2{\hbox {S}}^2$ maintains consistent convergence with regular residual decay while Plain-SC-TT exhibits non-convergent behavior characterized by irregular residual patterns. The final residual value of $\mathscr {O}(10^{-2})$ reflects the poor condition number of the linear system involved^19,24^. Moreover, the computational cost of ${\hbox {T}}^2{\hbox {S}}^2$ for solving a problem with $\#\textsf{dofs}=\mathcalligra{\scriptstyle O}(10^{9})$ on an Intel i9 laptop computer is in the range of seconds.

### Low-dimensional numerical challenges

This section presents two low-dimensional test cases to assess the characteristic capabilities of ${\hbox {T}}^2{\hbox {S}}^2$. The first test is the traveling bump benchmark^21,31^. The bump benchmark is a two-dimensional time-dependent problem where the initial condition is known, but there is no a priori knowledge of the evolution of the solution. The test is based on a rotational velocity flow, that challenges the Cartesian structure of the ${\hbox {T}}^2{\hbox {S}}^2$ grid. Although we choose collocation points to be the zeros of $P_n$, considering positive convection coefficients in both directions, ${\hbox {T}}^2{\hbox {S}}^2$ nonetheless proves to be robust. We solve problem (1)–(3) with ${\varvec \beta }=(-x_2,x_1)^T$, $\varepsilon =10^{-6}$ and $\rho = 0$. The space-time domain is given by $\Omega \times T=\left( [-1,1]\times [-1,1]\right) \times [0,\pi ]$. The initial condition is:

$$\begin{aligned} f(x_1,x_2,0) = \left\{ \begin{array}{ll} 16\,\left( 1-4\,\left( x_2-\frac{1}{2}\right) ^2\right) ^2\,\left( 1-4\,x_1^2\right) ^2 & x_2 > 0,\ \frac{1}{2} \le x_1 < \frac{1}{2}, \\ 0 & \mathrm {elsewhere.} \end{array} \right. \end{aligned}$$

**Fig. 8:**
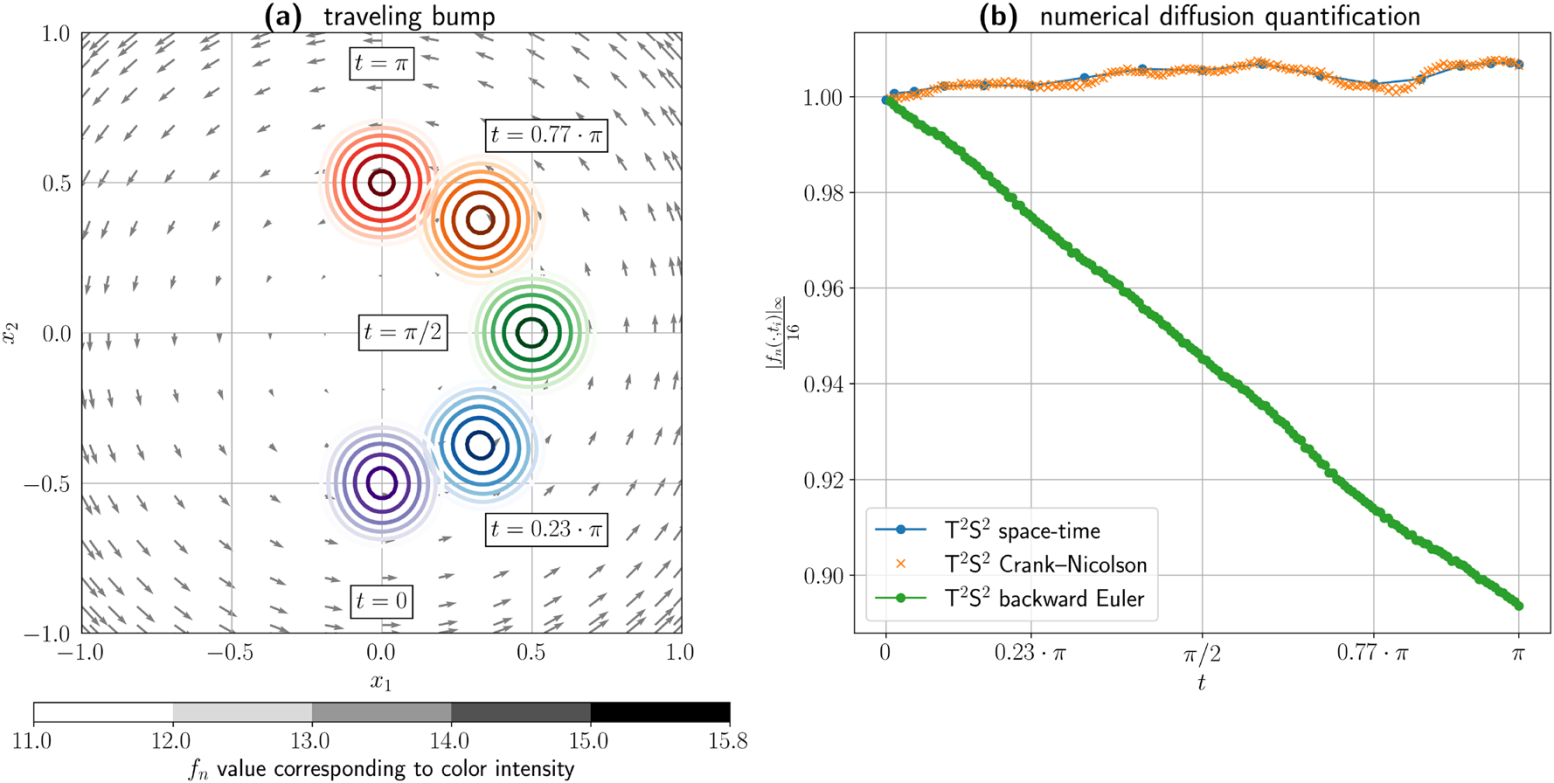
Results for the traveling “bump” test case. (**a**) Contour lines for the “bump” at different time steps. Different colors distinguish the different instants; the shades distinguish the different function values. The different levels are reported in the gray color bar. (**b**) The time evolution of the solution peak values for three ${\hbox {T}}^2{\hbox {S}}^2$ method’s implementations using the implicit backward Euler scheme, the semi-implicit Crank–Nicolson scheme, and the space-time formulation. A video of the traveling bump is provided as Supplementary Materials.

The polynomial degree of the basis functions is 16. Figure 8 presents the results of this calculation, in the case of the spacetime formulation. Panel ${(a)}$ illustrates the evolution of the bump’s position within the domain, which describes a circular trajectory through contour lines at various time snapshots, together with the prescribed velocity field. Theoretically, the configuration at time $\pi$ should be mirror symmetric versus the initial condition. Panel ${(b)}$ provides quantitative accuracy analysis through the normalized maximum norm $\Vert f_{n}(\cdot ,t_i)\Vert _\infty /16$, computed on a $128\times 128$ uniform grid. The results demonstrate that ${\hbox {T}}^2{\hbox {S}}^2$ preserves the solution structure with minimal oscillation behavior. In this panel we superimpose the result for the spacetime formulation and the time marching schemes: backward Euler and Crank-Nicolson. The implicit backward Euler scheme reads:

6 $$\begin{aligned} f^{k+1} - \Delta t\Big ( \varepsilon \Delta f^{k+1} - {\varvec \beta }\cdot \nabla f^{k+1} \Big ) = f^{k}, \end{aligned}$$

and the semi-implicit Crank–Nicolson scheme is:

7 $$\begin{aligned} f^{k+1} - \frac{\Delta t}{2}\Big ( \varepsilon \Delta f^{k+1} - {\varvec \beta }\cdot \nabla f^{k+1} \Big ) = f^{k} + \frac{\Delta t}{2} ( \varepsilon \Delta f^{k} - {\varvec \beta }\cdot \nabla f^{k} ). \end{aligned}$$

We choose a time step $\Delta t = \pi /160$ in both cases. Since the implicit backward Euler scheme is only first-order accurate, the solution approximation shows significative numerical diffusion as the peak’s value is reduced by roughly 10%. The Crank-Nicolson scheme is particularly well-suited for this problem due to its reduced numerical dissipation compared to other schemes. This property is critical as it better preserves the system’s physical quantities, which is a key characteristic in many applicative problems.

**Fig. 9:**
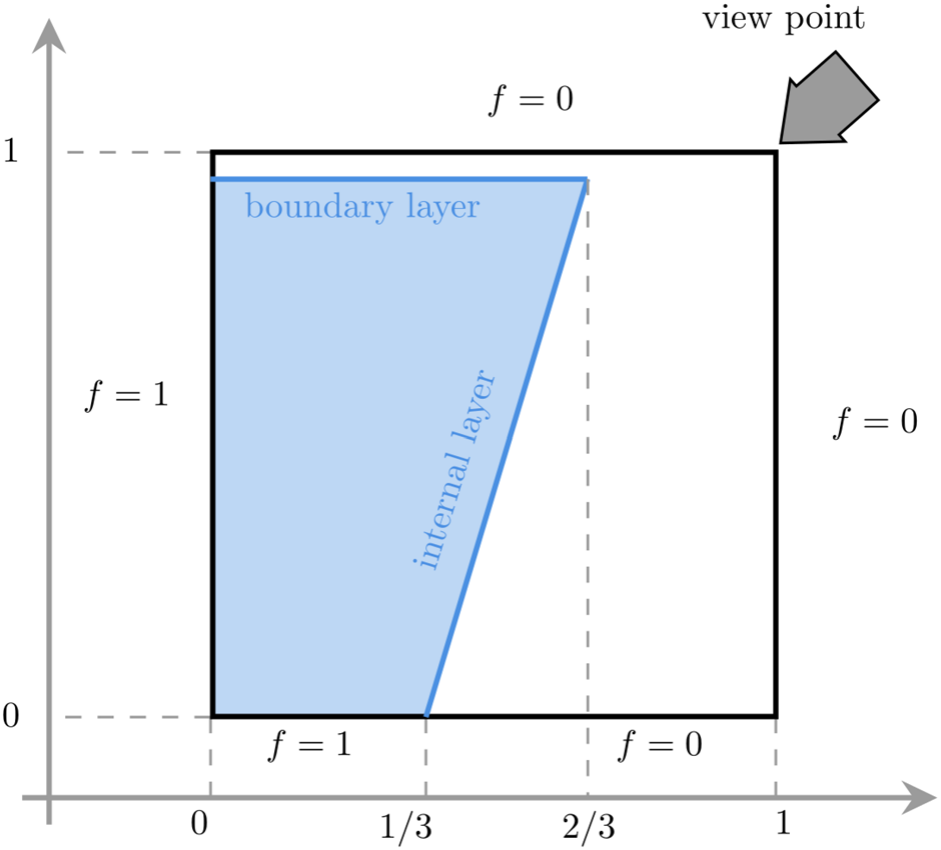
This picture gives a schematic overview of the convection-diffusion test case as proposed by Hughes and coauthors^32,33^, highlighting the boundary and internal layers of the solution.

**Fig. 10:**
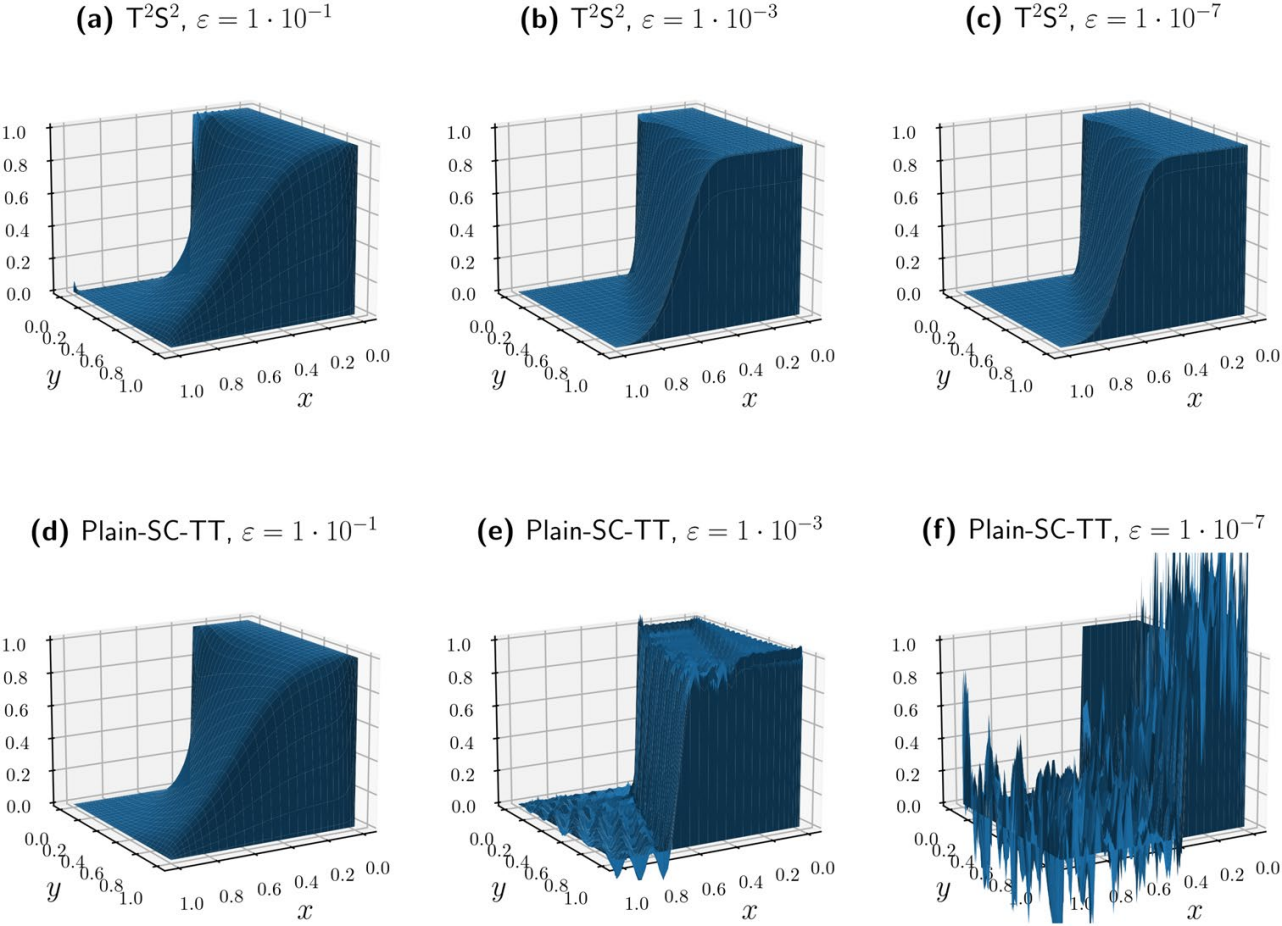
This picture represents the solutions of the test proposed by Hughes and coauthors^32,33^ for various values of the parameter $\varepsilon$ and for the Plain-SC-TT collocation method versus ${\hbox {T}}^2{\hbox {S}}^2$. The solution degree is 63. While Plain-SC-TT shows spurious oscillations at $\varepsilon =10^{-3}$, ${\hbox {T}}^2{\hbox {S}}^2$ is stable regardless of the value of $\varepsilon$.

The second test case, originally proposed by Tom Hughes and coauthors^32,33^, evaluates the method’s ability to resolve both boundary and internal layers. We solve problem (5) in two dimensions with $b=0$ and decreasing values of $\varepsilon$. The discontinuous boundary at $x_2=0$, which is transported in the interior by the convection field ${\varvec \beta }=(1,3)^T$, generates the internal layer on the bottom side of the computational domain. The boundary layer is determined by setting a homogeneous boundary condition on the top side of the computational domain. The test case specifics are sketched in Fig. 9. The boundary layer connects the solution with the boundary conditions and the internal layer connects two flat regions of the solution, i.e., $f=1$ (on the left), and $f=0$ (on the right). The reader will notice that the smaller is $\varepsilon$, the higher is the gradient of the internal layer; see Figs. 10a–c. ${\hbox {T}}^2{\hbox {S}}^2$ method confirms its oscillatory-free behavior. In contrast, Plain-SC method exhibits spurious numerical oscillations, Fig. 10d–f, which start being visible for a relatively big value of $\varepsilon =10^{-3}$, and fails to resolve the problem accurately.

## Discussion

${\hbox {T}}^2{\hbox {S}}^2$ effectively integrates the strengths of its core components: Spectral Collocation for accuracy, Superconsistency for stability in the presence of boundary or internal layers, and TT format as a low-rank linear algebra framework. In Section “Results”, we demonstrated that ${\hbox {T}}^2{\hbox {S}}^2$ reduces the approximation error to the range of $\mathcalligra{\scriptstyle O}^{-12}$, with the error decreasing sharply when plotted on a logarithmic scale. Despite using a low-rank solution, the grid density is such that traditional solvers—even at theoretically optimal efficiency would require millions of years on the current world’s most powerful architectures. ${\hbox {T}}^2{\hbox {S}}^2$ preserves spectral accuracy in high-dimensional, high dense grid scenarios. In the section about stability, we tested the solver’s stability using a six-dimensional stationary test case characterized by a boundary layer solution, i.e., a solution characterized by a high gradient region connecting the domain interior to the homogeneous boundary conditions. This test not only confirms the unconditional stability of the method versus the parameters choice, but also shows that ${\hbox {T}}^2{\hbox {S}}^2$ can approximate high dimensional/high gradient solutions with a rank in the range of 10. ${\hbox {T}}^2{\hbox {S}}^2$ achieves this without requiring any intermediate approximation layers, either at the continuous or discrete level: the discrete version of problem (1) fits naturally into the TT structure. Moreover, ${\hbox {T}}^2{\hbox {S}}^2$ differs fundamentally from low-rank data-driven approaches, such as machine learning and reduced-order modeling^41,42^ as it does not require any high-fidelity offline computations or training phases. As discussed in “Results” , we remarked that a high-fidelity computation in seven dimensions results in prohibitive computational costs, even for a relatively coarse grid. Following the characterization of low-rank methods proposed in the comprehensive review by Einkemmer et al.^12^, a time discretization of ${\hbox {T}}^2{\hbox {S}}^2$, such as the backward Euler and Crank-Nicolson schemes, falls into the category of the Step And Truncate (SAT) methods. SAT methods offer the flexibility to incorporate ${\hbox {T}}^2{\hbox {S}}^2$. Alternatively, sparse grid methods represent a worthmentioning attempt to tackle the course of dimensionality. They are capable of reducing the total number of degrees of freedom to $\mathcalligra{\scriptstyle O}{(\#\textsf{dofs}_{\textrm{1d}} \cdot \log (\#\textsf{dofs}_{\textrm{1d}})^{d-1})}$, possibly lower compression ratios can be achieved through adaptivity^43^. While this is a remarkable result, considering a grid with a number of nodes in the order of $100^7 = 10^{14}$ and solution rank around ten, sparse grids still need $10^8$ degrees of freedom, while ${\hbox {T}}^2{\hbox {S}}^2$ can compress the data from $10^{14}$ to $7\cdot 10^4$, without compromising for accuracy. Low-rank approximation covers a wide variety of practical problems. However even if this prerogative is not satisfied, our method can still be effectively integrated to approximate initial solution guesses or as a preconditioner. Regular grids are another commonly highlighted limitation of TT technology. Even though this limitation holds true, ${\hbox {T}}^2{\hbox {S}}^2$ strongly cuts computational costs enabling much denser grids than standard discretisations. This ${\hbox {T}}^2{\hbox {S}}^2$ capability also nicely couples with immersed methods like immersed boundary method^44^, fictitious domain^45^, or distributed Lagrangian multiplier^46^. These techniques are valuable tools to enforce non-standard boundary conditions or geometries.

Problem (1) serves as the foundation for a wide range of solvers in fields such as radiation transport physics, kinetic theory, and computational fluid dynamics. As a result, ${\hbox {T}}^2{\hbox {S}}^2$ is a tool for a wide range of applications in modeling high-dimensional transport phenomena. Technological advances are driving the development of next-generation nuclear technologies, including small modular reactors, advanced modular reactors, microreactors, and nuclear fusion systems. These innovations demand large-scale neutron transport and fluid dynamics calculations for reactor design and throughout the nuclear fuel cycle, including nuclear safeguards, nuclear non-proliferation, and security needs^47–51^. For istance, the Boltzmann neutron transport equation, which neglects neutron-neutron collisions, is a linear equation. Similarly, the Vlasov equation, used in plasma physics, is also a linear equation where the non-linearity comes from the coupling with Maxwell’s equations. The same, linear transport equations govern photon transport. We anticipate ${\hbox {T}}^2{\hbox {S}}^2$ to be a key numerical solver in addressing these complex computational challenges. The applications are not limited to linear problems only. In the kinetic theory of gases, in computational fluid dynamics and computational chemistry^52,53^, the transport equations often involve nonlinear terms, whose solution strategy can be approximated by solving a sequence of linear problems, an approach that can be addressed using the ${\hbox {T}}^2{\hbox {S}}^2$ method. In summary, ${\hbox {T}}^2{\hbox {S}}^2$ has the potential to impact a wide range of fields requiring efficient, high-dimensional transport computations. Its ability to solve such problems on standard computing hardware makes it particularly valuable for practical applications with limited computational resources. Future directions include extending the ${\hbox {T}}^2{\hbox {S}}^2$ architecture to run efficiently on supercomputers, expanding its applicability to large-scale scientific and engineering problems.

## Methods

In this section, we provide a formal mathematical description of the ${\hbox {T}}^2{\hbox {S}}^2$ scheme. We introduce the one-dimensional operators in the subsection “Spectral Collocation Methods”. Next, in section “TT representation” we introduce the TT linear algebra format. Finally, in the section “${\hbox {T}}^2{\hbox {S}}^2$algorithm for convection-diffusion-reaction equation” we present how the building blocks are integrated to construct the high-dimensional solver.

### Spectral collocation methods

Spectral approximations. Let $\mathcalligra{\scriptstyle X}\:$ and $\mathcalligra{\scriptstyle Y}\:$ be suitable functional spaces. We consider a linear operator $L:\mathcalligra{\scriptstyle X}\:\rightarrow\mathcalligra{\scriptstyle Y}\:$ and, for $b\in\mathcalligra{\scriptstyle Y}\:$, we are interested in solving the problem

8 $$\begin{aligned} \text{ Find } f\in \mathcalligra{\scriptstyle X}\:\: \text{ such } \text{ that: }\quad L\,f= b. \end{aligned}$$

The operator $L$ can be rather generic, but for this paper, we only examine applications in the framework of transport-diffusion PDEs. In this case, formulation (8) includes boundary and initial conditions to be assigned to $f$. We assume that problem (8) is mathematically well-posed in the sense of Hadamard^54^, and we numerically discretize it through the spectral collocation method based on algebraic polynomials. Spectral methods are a very effective tool in numerical approximation of various mathematical models due to their excellent convergence rate in the presence of very smooth solutions^19,21^.

We express $f$ as a linear combination of $n+1$ basis functions denoted by $l_{i}(x)$, $0\le i\le n$. These functions form a polynomial Lagrangian basis for a predefined set of nodes $\vec {\bf{x}}=\{x_{j}\}_{j=0,1,\ldots ,n}$ in the computational domain, so that $l_{i}(x_{j})=\delta _{ij}$. The points $x_{i}$ are the *collocation points* and form the *collocation grid*. In this way, we will assume that $f(x)\approx f_{n}(x)=\sum _{0\le i\le n}f_{i}\ l_{i}(x)$. As a consequence $f_{n}\in \mathcalligra{\scriptstyle X}_{n}$, where $\mathcalligra{\scriptstyle x}_{n}$ is a finite dimensional space.

In the examples to follow, the distribution of such nodes is not uniform (i.e., with fixed mesh size). Indeed, we set $x_{0}=-1$ and $x_{n}=1$, and the remaining nodes are the zeros of $P_{n}'$, which is the first derivative of the $n$-th Legendre polynomial $P_{n}$.

In the pseudospectral method, Eq. (8) is required to be satisfied at the collocation nodes $\vec {\bf{x}}$. To fix the idea, let us take $L:=d^2/{dx^2}$ and let $l_{i}''$ be the second derivative of each element of the basis function $l_{i}$, for $i=0,1,\ldots ,n$, so that $f_{n}''(x)=\sum _{0\le i\le n}f_{i}\ l_{i}''(x)$ (note that there is no approximation here since the second derivative of a polynomial can be computed exactly). We then build a corresponding linear system, that, by choosing for instance $n=4$, takes the form:

9 $$\begin{aligned} \big (L_{n}f_{n}\big )(\vec {\bf{x}}):={\underbrace{ { \left[ \begin{array}{ccccc} l_{0}''(x_0) & l_{0}''(x_1) & l_{0}''(x_2) & l_{0}''(x_3) & l_{0}''(x_4) \\[0.25em] l_{1}''(x_0) & l_{1}''(x_1) & l_{1}''(x_2) & l_{1}''(x_3) & l_{1}''(x_4) \\[0.25em] l_{2}''(x_0) & l_{2}''(x_1) & l_{2}''(x_2) & l_{2}''(x_3) & l_{2}''(x_4) \\[0.25em] l_{3}''(x_0) & l_{3}''(x_1) & l_{3}''(x_2) & l_{3}''(x_3) & l_{3}''(x_4) \\[0.25em] l_{4}''(x_0) & l_{4}''(x_1) & l_{4}''(x_2) & l_{4}''(x_3) & l_{4}''(x_4) \end{array} \right] } }_{{{D^2}}} } \left[ \begin{array}{c} f_{n}(x_0) \\[0.25em] f_{n}(x_1) \\[0.25em] f_{n}(x_2) \\[0.25em] f_{n}(x_3) \\[0.25em] f_{n}(x_4) \end{array} \right] = \left[ \begin{array}{c} b(x_0) \\[0.25em] b(x_1) \\[0.25em] b(x_2) \\[0.25em] b(x_3) \\[0.25em] b(x_4) \end{array} \right] =:b(\vec {\bf{x}}). \end{aligned}$$

In Eq. (9), the left-hand side matrix, called $D^2$, provides the spectral collocation discretization of the second derivative operator $d^2/{dx^2}$. The entries of such a matrix are constructed by evaluating the second derivatives of each element of the Lagrange basis at the collocation points. For a general *n*, we have that $D^2=L_{n}=\{l_{i}''(x_{j})\}$, $i,j=0,1,\ldots ,n$, is a $(n+1)\times (n+1)$ matrix. To impose Dirichlet boundary conditions, the first and the last row of the above matrix will be forced to be the corresponding first and last row of the $(n+1)\times (n+1)$ identity matrix. Furthermore, we impose the proper boundary conditions by suitably replacing $b(x_0)$ and $b(x_4)=b(x_n)$. For example, we set $b(x_0)=b(x_n)=0$ in the homogeneous case.

The theory of stability and convergence of the spectral collocation method dates back to the 70s^19,55^. We briefly recall that stability requires the boundedness of the approximate solution in some suitable norm. The bound must be uniform versus the discretization parameter *n*. As the consistency property is satisfied, i.e., exact and discretized operators coincide in a suitable finite dimensional space, convergence of $f_{n}$ to $f$ is obtained in certain normed spaces^19^. The convergence, for spectral type approximations, displays an exponential rate under the hypotheses of high regularity of the solution^56^. This makes this numerical technique very competitive with other known methods, as it requires far fewer degrees of freedom if the regularity assumptions on the exact solution are fulfilled.

Superconsistency to define a superconsistent method, we introduce a second grid of $n-1$ points, e.g., $\vec {\bf{z}}=\{z_{j}\}_{j=1,\ldots ,n-1}$, within the computational domain, according to the following recipe. Hereafter, we will refer to the points $x_{i}$ introduced in the previous section as the *representation points*, and the points $z_{i}$ as the *collocation points*. Accordingly, we say that $\vec {\bf{x}}$ is the *representation grid*, and $\vec {\bf{z}}$ is the *collocation grid*. We use the representation points to express $f_{n}=\{ f_{n}(x_i) \}$. Instead, we use the collocation points to derive the numerical method. The approximation still keeps the form introduced above, but now we require that $L_{n}(f_{n}(\vec {\bf{x}}))=\big (L_{n}f_{n}\big )(\vec {\bf{z}})=b_{n}(\vec {\bf{z}})$. Now $L_{n}$ transforms a vector built at the nodes $\vec {\bf{x}}$ into a vector built at the nodes $\vec {\bf{z}}$. Thus, at the interior of the domain, the new set of points replaces the old one, which is only used to express the approximated solution. In other words, we look for the values $f_{n}(\vec {\bf{x}})$, but we require the problem to be satisfied at the points $\vec {\bf{z}}$. The two sets of points may coincide, but this depends on the operator $L$, to realize a specific property. The method is said to be *superconsistent* if the following consistency condition holds for a functional space $\widetilde{\mathcalligra{\scriptstyle X}}\:_{n}$ larger than $\mathcalligra{\scriptstyle X}\:_{n}$:

10 $$\begin{aligned} \big (Lq\big )(\vec {\bf{z}}) = L_{n}\big (q(\vec {\bf{x}})\big ) \qquad \forall q\in \widetilde{\mathcalligra{\scriptstyle X}}\:_{n}. \end{aligned}$$

This means that the exact operator applied to $q$ and successively evaluated at $\vec {\bf{z}}$, equals the discrete operator applied to the same $q$ evaluated at the nodes $\vec {\bf{x}}$. We want this to be true for functions that are outside the approximation space $\widetilde{\mathcalligra{\scriptstyle X}}\:_{n}$, realizing in this way a higher degree of consistency. This construction can be done, in principle, for a large range of problems, as, for example, the approximation of integral equations^57^.

Let $\widetilde{\mathcalligra{\scriptstyle X}}\:_{n}={\mathcalligra{\scriptstyle X}}\:_{n}\cup \text {span}(\chi )$ and consider the one-dimensional convection-diffusion operator

$$\begin{aligned} L:=\beta \cdot d/{dx} - \varepsilon \cdot d^2/{dx^2}. \end{aligned}$$

We consider the function $\chi :=\chi _{n+1}=(1-x^2)\,P_{n}'$, where $P_{n}'$ is the first derivative of the Legendre polynomial $P_{n}$ of degree *n*^58^. Now, imposing the superconsistent condition amounts to determining the points $\vec {\bf{z}}$ in such a way that:

$$\begin{aligned} n(n+1) \left[ \varepsilon P_{n}' - \beta P_{n} \right] (z_{j}) = 0\ \quad \textrm{for}\,j=1,\ldots ,n-1. \end{aligned}$$

These points are located between the zeros of $P_{n}$ and $P_{n}'$ and their location depends on the ratio between $\varepsilon$ and $\beta$, as shown in Fig. 1. We denote $S_n$ the $(n+1)\times (n+1)$ interpolation matrix that maps values at points $\vec {\bf{x}}$ to values at points $\vec {\bf{z}}$. If $L_n$ is the discrete operator evaluated at the representation points, $S_n \cdot L_n$ is the operator evaluated at the collocation points.

### TT representation

We first review some basic notation and definitions about tensors^59^ and, in particular, the TT format^13,60^. Then, we review some important basic facts about the TT decomposition.

Notation and generalities.

A $d$-dimensional tensor $\mathcalligra{\scriptstyle A}=\big \{\mathcalligra{\scriptstyle A}\:(i_{1},i_{2},\ldots ,i_{d})\big \},\,i_{\ell }=1,2,\ldots ,n_{\ell },\,\ell =1,2,\ldots ,d$ is a multi-dimensional array whose entries are indexed by $d$ indices. We denote $d$-dimensional tensors using calligraphic, upper case fonts, such as $\mathcalligra{\scriptstyle A},\mathcalligra{\scriptstyle B},\mathcalligra{\scriptstyle C}$. For scalars, vectors, and matrices, which are formally $d$-way tensors with, respectively, a number of dimensions $d=0,1,2$, we adopt a different notation for clarity. Precisely, we denote scalar quantities using normal, lower-case fonts as in $a,b,c$; vector fields using bold, lower-case fonts as in ${\bf{a}},{\bf{b}},{\bf{c}}$; matrices using normal, upper case fonts as in $A,B,C$. Furthermore, we denote the entries of a vector ${\bf{a}}$ as $a(i)$ and the entries of a matrix $A$ as $A(i,j)$. We may occasionally denote the entries of three-dimensional tensor as $\mathcalligra{\scriptstyle A}\:(i,j,k)$ instead of $\mathcalligra{\scriptstyle A}\:(i_{1},i_{2},i_{3})$, but we prefer using the notation $\mathcalligra{\scriptstyle A}\:(i_{1},i_{2},\ldots ,i_{d})$ when $d>3$. For completeness, we anticipate that when we consider a three-dimensional tensor that is also a core of a TT decomposition (see below for the definition), the first and last indices are a Greek letter, e.g., $\alpha , \beta$ etc, and the intermediate index is always a Roman letter, e.g., *i*, *j* etc.

Following a MATLAB-style notation, we denote free indices using the symbol “ : ”. This notation makes it possible to represent the two important structural concepts of *fiber* and *slice* of a tensor. A fiber is a vector obtained by fixing all but one index, and a slice is a matrix obtained by fixing all but two indices. For example, a 3D tensor $\mathcalligra{\scriptstyle A}\:(i,j,k)$ has three different types of fibers, i.e., $\mathcalligra{\scriptstyle A}\:(:,j,k)$, $\mathcalligra{\scriptstyle A}\:(i,:,k)$, $\mathcalligra{\scriptstyle A}\:(i,j,:)$, and three different types of slices $\mathcalligra{\scriptstyle A}\:(i,:,:)$, $\mathcalligra{\scriptstyle A}\:(:,j,:)$, $\mathcalligra{\scriptstyle A}\:(:,:,k)$. Finally, always according with the MATLAB-style notation, a repetition of the free index symbol “ : ” denotes an index contraction. For example, let $A$, $B$, $C$ be three matrices with compatible sizes such that $C=AB$. Then, the notation “$C(i,j)=A(i,:)B(:,j)$” is equivalent to $C(i,j)=\sum _{\alpha }A(i,\alpha )B(\alpha ,j)$, where the summation over the index $\alpha$ is taken, as usual, over all the elements of the *i*-th rows of $A$ and *j*-th columns of $B$.

TT format.

The TT format provides an efficient representation of high-dimensional tensors by decomposing them into a product of much smaller, three-dimensional tensors called the “TT cores”. Specifically, a $d$-dimensional tensor $\mathcalligra{\scriptstyle A}\:\in \mathbbm {R}^{n_{1}\times \cdots \times n_{d}}$ is said to be in TT format if there exist $d$ cores $\mathcalligra{\scriptstyle A}\:_{\ell } \in \mathbbm {R}^{r_{\ell -1}\times n_{\ell }\times r_{\ell }}$, for $\ell =1,\ldots ,d$, with $r_{0}=r_{d}=1$, such that each entry of $\mathcalligra{\scriptstyle A}\:$ can be computed as:

11 $$\begin{aligned} \mathcalligra{\scriptstyle A}\:(i_{1},\ldots ,i_{d}) = \sum _{\alpha _1=1}^{r_{1}}\cdots \sum _{\alpha _{d-1}=1}^{r_{d-1}} \mathcalligra{\scriptstyle A}\:_{1}(1,i_{1},\alpha _1)\mathcalligra{\scriptstyle A}\:_{2}(\alpha _1,i_{2},\alpha _2)\cdots \mathcalligra{\scriptstyle A}\:_{d}(\alpha _{d-1},i_{d},1). \end{aligned}$$

In some sense, Eq. (11) is a multi-dimensional generalization of the (reduced) Singular Value Decomposition (SVD) of matrices. Note, however, that we do not require any specific orthogonality property in the above definition (more details on the SVD connection will be given in the next example and later in this section).

**Fig. 11:**
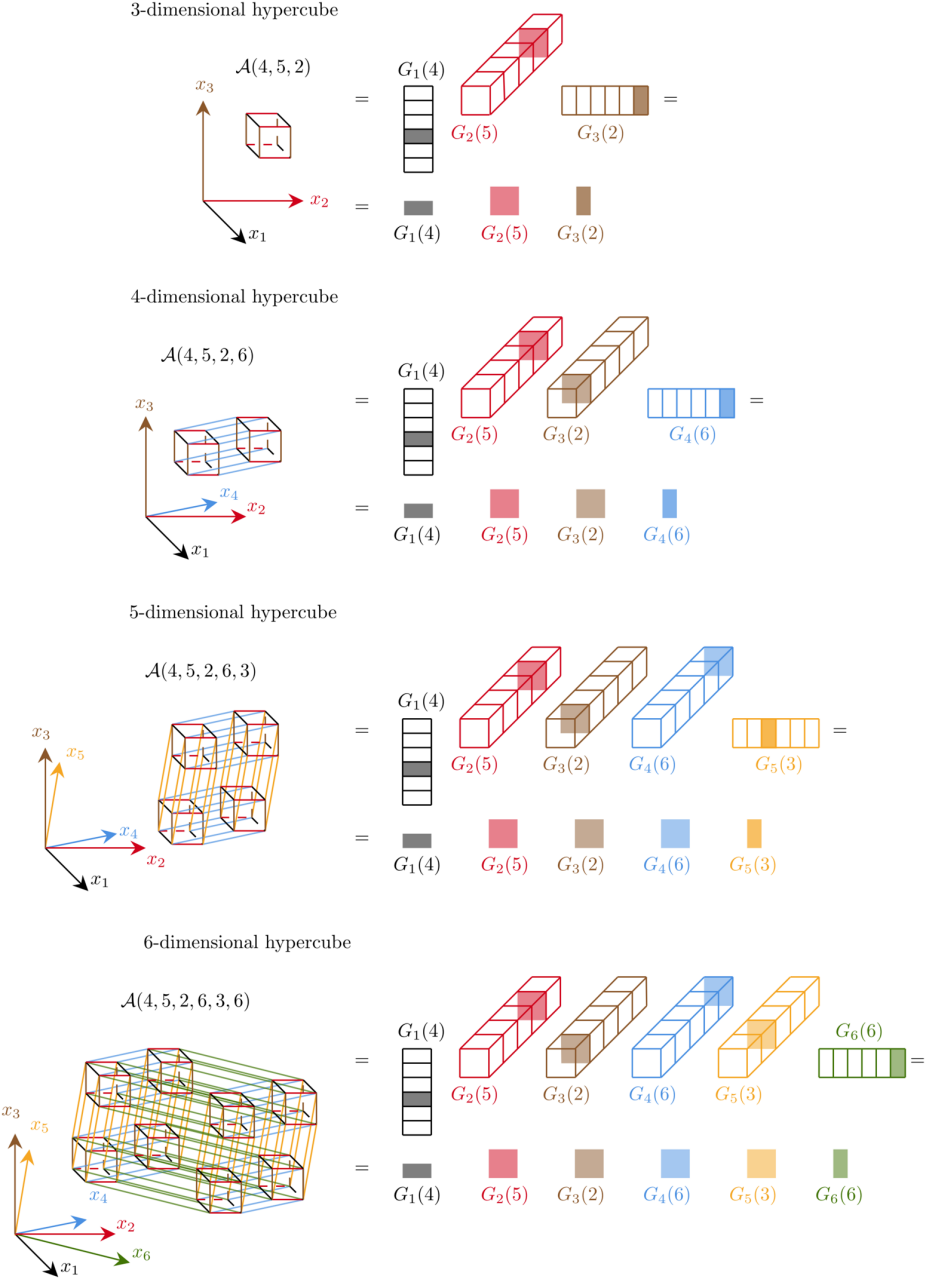
In this picture we present the progression from a three to a six-dimensional array in full and TT format.

To fix ideas, consider the following two dimensional case. A matrix $A\in \mathbbm {R}^{n_{1}\times n_{2}}$ is decomposed in the product of two matrices $A_{1}\in \mathbbm {R}^{n_{1}\times r_{1}}$ and $A_{2}\in \mathbbm {R}^{r_{1}\times n_{2}}$, so that $A(i_{1},i_{2})=\sum _{\alpha =1}^{r_{1}}A_{1}(i_{1},\alpha )A_{2}(\alpha ,i_{2})$. We can easily construct such a decomposition through the SVD of $A$, which we write as usual as $A=U\Sigma V^T$, where $U\in \mathbbm {R}^{n_{1}\times r_{1}}$ and $V\in \mathbbm {R}^{n_{2}\times r_{1}}$ are two orthogonal-by-column matrices, and $\Sigma \in \mathbbm {R}^{r_{1}\times r_{1}}$ is the diagonal matrix with $r_{1}$ strictly positive singular values, so that $r_{1}$ is the rank of matrix $A$. Then, take, for instance, $A_{1}=U$, $A_{2}=\Sigma V^T$, or $A_{1}=U\Sigma$, $A_{2}=V^T$. We can also use the tensor notation introduced above and specialized to the case $d=2$, so that we can write $\mathcalligra{\scriptstyle A}\:(i_{1},i_{2})=\sum _{\alpha =1}^{r_{1}}\mathcalligra{\scriptstyle A}\:_{1}(1,i_{1},\alpha )\mathcalligra{\scriptstyle A}\:_{2}(\alpha ,i_{2},1)$, where $\mathcalligra{\scriptstyle A}\:_{1}\in \mathbbm {R}^{r_{0}\times n_{1}\times r_{1}}$, $\mathcalligra{\scriptstyle A}\:_{2}\in \mathbbm {R}^{r_{1}\times n_{2}\times r_{2}}$, with $r_{0}=r_{2}=1$. The decomposition process can be easily extended to $d=3$ by redefining $\mathcalligra{\scriptstyle A}\:_{2}$ as the intermediate core $\mathcalligra{\scriptstyle A}\:_{2}\in \mathbbm {R}^{r_{1}\times n_{2}\times r_{2}}$ and introducing a final core $\mathcalligra{\scriptstyle A}\:_{3}\in \mathbbm {R}^{r_{2}\times n_{3}\times r_{3}}$ with $r_{3}=1$, so that $\mathcalligra{\scriptstyle A}\:(i_{1},i_{2},i_{3}) = \sum _{\alpha _1=1}^{r_{1}}\sum _{\alpha _2=1}^{r_{2}} \mathcalligra{\scriptstyle A}\:_{1}(1,i_{1},\alpha _1)\mathcalligra{\scriptstyle A}\:_{2}(\alpha _1,i_{2},\alpha _2)\mathcalligra{\scriptstyle A}\:_{2}(\alpha _2,i_{3},1)$. This process can be continued by adding one new dimension at each step as it is illustrated in Fig. 11 for the construction of the TT representation of a tensor up to $d=6$.

**Remark.** The computational effectiveness of the TT representation of multi-dimensional arrays is related to controlling the core ranks. To exemplify this fact, consider a six-dimensional array with mode sizes $n=n_{1}=\ldots =n_{6}=10^3$ and assume that the ranks are all equal to $r=r_{1}=\ldots =r_{d-1}=10$. In such a case, the total storage required to represent such a tensor in TT format is proportional to $\mathscr {O}(nr^2d)=6\,10^5$ against a full grid storage requirement of $\mathscr {O}(n^d)=10^{18}$.

The TT representation can equivalently be reformulated in a more compact form by representing each core as a set of slice matrices parametrized with the spatial index $i_{\ell }$, e.g., $A_{\ell }(i_{\ell })=\mathcalligra{\scriptstyle A}\:_{\ell }(:,i_{\ell },:)\in \mathbbm {R}^{r_{\ell -1}\times r_{\ell }}$, yielding:

12 $$\begin{aligned} \mathcalligra{\scriptstyle A}\:(i_{1},\ldots ,i_{d}) = A_{1}(i_{1})A_{2}(i_{2})\cdots A_{d}(i_{d}). \end{aligned}$$

The internal sizes of the decomposition, i.e., the set of $(d-1)$ integers $\{r_{\ell }\}_{\ell =1}^{d-1}$, are the the *TT-ranks*, or, simply, the *ranks*, of the TT decomposition. A convenient upper bound on them is their maximum value, which we denote as $r=\max _{\ell }\{r_{\ell }\}$. We also introduce an upper bound on the dimensions, denoted as $n=\max _{\ell }n_{\ell }$. The TT-ranks determine the storage complexity of the representation, which scales linearly with the dimension $d$ as $\mathscr {O}(dnr^2)$, in contrast to the exponential growth $\prod _{\ell =1}^{d}n_{\ell }=\mathscr {O}(n^{d})$ required for the full tensor representation. The linear dependence on $d$ of the TT-representation of a tensor is very convenient, reflecting the compression efficiency of such a representation, whenever $r\ll n$. In such a case, we say that (11) and (12) are a *low-rank representation* of tensor $\mathcalligra{\scriptstyle A}\:$.

If we introduce the multi-index $i=(i_{1},i_{2},\ldots ,i_{d})$, we can think that Eq. (11) defines the TT format representation for a sort of “*multi-dimensional vector*”, i.e., $\mathcalligra{\scriptstyle A}\:(i)$. It is also possible to adopt a similar *TT format for linear operators*, i.e., “*multi-dimensional matrices*” $\mathcalligra{\scriptstyle L}\:(i,j)$ that depend on two $d$-dimensional multi-indices $i=(i_{1},i_{2},\ldots ,i_{d})$ and $j=(j_{1},j_{2},\ldots ,j_{d})$. The formal definition reads as

13 $$\begin{aligned}&\mathcalligra{\scriptstyle L}\:\Big (\big (i_{1},i_{2},\ldots ,i_{d}\big ),\,\big (j_{1},j_{2},\ldots ,j_{d}\big )\Big ) =\mathscr {L}\Big (\big (i_{1},j_{1}\big ),\big (i_{2},j_{2}\big ),\ldots ,(i_{d},j_{d}\big )\Big )\nonumber \\&\hspace{2cm} = \sum _{\alpha _1=1}^{r_{1}}\sum _{\alpha _2=1}^{r_{2}}\ldots \sum _{\alpha _{d-1}=1}^{r_{d-1}} L_{1}\big (1,(i_{1},j_{1}),\alpha _1\big )\, L_{2}\big (\alpha _2,(i_{2},j_{2}),\alpha _3\big )\, \ldots L_{d}\big (\alpha _{d-1},(i_{d},j_{d}),1\big ), \end{aligned}$$

where the first equality follows from a convenient index permutation to form the index pairs $(i_{\ell },j_{\ell })$, $\ell =1,2,\ldots ,d$.

The so-called “*core notation*” gives a more intuitive insight into the structure of the cores:

$$\begin{aligned} A_{\ell }= \left[ \begin{array}{cccc} A_{\ell }(1,:,1) & A_{\ell }(1,:,2) & \ldots & A_{\ell }(1,:,r_{\ell }) \\ A_{\ell }(2,:,1) & A_{\ell }(2,:,2) & \ldots & A_{\ell }(2,:,r_{\ell }) \\ \vdots & \vdots & \vdots & \vdots \\ A_{\ell }(r_{\ell -1},:,1) & A_{\ell }(r_{\ell -1},:,2) & \ldots & A_{\ell }(r_{\ell -1},:,r_{\ell })\\ \end{array} \right] ,\quad \end{aligned}$$

$$\begin{aligned} L_{\ell }= \left[ \begin{array}{cccc} L_{\ell }(1,:,:,1) & L_{\ell }(1,:,:,2) & \ldots & L_{\ell }(1,:,:,r_{\ell }) \\ L_{\ell }(2,:,:,1) & L_{\ell }(2,:,:,2) & \ldots & L_{\ell }(2,:,:,r_{\ell }) \\ \vdots & \vdots & \vdots & \vdots \\ L_{\ell }(r_{\ell -1},:,:,1) & L_{\ell }(r_{\ell -1},:,:,2) & \ldots & L_{\ell }(r_{\ell -1},:,:,r_{\ell })\\ \end{array} \right] . \end{aligned}$$

With such notation and using the definition of the strong Kroncker product^61^ “$\bowtie$”, we write the TT decomposition in the more compact form:

$$\begin{aligned} \mathcalligra{\scriptstyle A}\: = A_{1}\bowtie A_{2}\bowtie \ldots \bowtie A_{d},\qquad \mathcalligra{\scriptstyle L}\: = L_{1}\bowtie L_{2}\bowtie \ldots \bowtie L_{d}. \end{aligned}$$

We will make use of this mathematical formalism to describe the different parts of ${\hbox {T}}^2{\hbox {S}}^2$.

TT decomposition. The construction of an optimal TT representation for a $d$-dimensional tensor can be achieved through the TT-SVD algorithm^13^. This algorithm builds the TT decomposition through a systematic application of singular value decompositions on specific matrix representations of the tensor, known as *unfoldings*. For a $d$-dimensional tensor $\mathcalligra{\scriptstyle A}\:$, we consider $d-1$ “fundamental” matrix unfoldings. The $\ell$-th unfolding, denoted as $A_\ell$, reorganizes the tensor entries into a matrix of size $(\prod _{k=1}^\ell n_{k})\times (\prod _{k=\ell +1}^dn_{k})$ through the relation:

14 $$\begin{aligned} A_\ell ( \eta _\ell (i_{1},\ldots ,i_{\ell }), \eta '_\ell (i_{\ell +1},\ldots ,i_{d})) = \mathcalligra{\scriptstyle A}\:(i_{1},\ldots ,i_{d}), \end{aligned}$$

where $\eta _\ell$ and $\eta '_\ell$ are two bijective mappings between the respective index tuples, e.g., $(i_{1},\ldots ,i_{\ell })$ and $(i_{\ell +1},\ldots ,i_{d})$, often called “long indices”, and the row and column indices of matrix $A_\ell$. A very strong connection exists between the TT-ranks of tensor $\mathcalligra{\scriptstyle A}\:$ and the ranks of the unfolding matrices. In fact, the rank of the $\ell$-th matrix unfolding is a lower bound for the TT-rank $r_{\ell }$, and a constructive proof shows that TT-SVD can compute an *exact* TT-representation with TT-ranks $r_{\ell }$ equal to the ranks of the matrix unfoldings $A_\ell$^13^, Theorem 2.1, i.e., $r_{\ell }=\operatorname {rank}(A_{\ell })$ for $\ell =1,\ldots ,d-1$.

Of particular practical importance is the existence of low-rank approximations. The sequential nature of the TT-SVD algorithm ensures that the TT ranks are optimal in the sense that they provide the best possible approximation for a given accuracy threshold. If we truncate the singular values in the TT-SVD algorithm below a given threshold $\tau$, the resulting approximation error in the Frobenius norm is bounded by $\tau \sqrt{d-1}\Vert \mathcalligra{\scriptstyle A}\:\Vert _F$. Such an approximate TT decomposition keeps the TT ranks minimal while achieving the accuracy prescribed by $\tau$. Therefore, when computational constraints or data compression requirements need smaller ranks $r_{\ell }'\le \operatorname {rank}(A_{\ell })$, a best approximation $\mathcalligra{\scriptstyle A}\:^{TT}_{\text {best}}$ in the TT format is guaranteed to exist.

The TT-SVD algorithm efficiently provides a quasi-optimal approximation $\mathcalligra{\scriptstyle A}\:^{TT}$ in the sense that:

15 $$\begin{aligned} \Vert \mathcalligra{\scriptstyle A}\:- \mathcalligra{\scriptstyle A}\:^{TT}\Vert _{\mathcalligra{\scriptstyle F}}\: \le \sqrt{d-1} \inf _{\mathcalligra{\scriptstyle B}\:^{TT}\in \mathbbm{T}\mathbbm{T}({\bf{r}}'_\ell )} \Vert \mathcalligra{\scriptstyle A}\:- \mathcalligra{\scriptstyle B}\:^{TT}\Vert _{\mathcalligra{\scriptstyle F}}\:. \end{aligned}$$

Here, $\mathbbm{T}\mathbbm{T}({\bf{r}}'_\ell )$ denotes the manifold of TT representations with prescribed rank ${\bf{r}}'_\ell = (r'_1,\ldots ,r'_{d-1})$, and the multiplicative factor $\sqrt{d-1}$ represents the stability constant of the algorithm^60^. This quasi-optimality result establishes TT-SVD as a practical tool for tensor approximation, offering a controlled trade-off between computational efficiency and approximation accuracy.

The TT format supports the efficient implementation of fundamental multi-linear algebraic operations such as scalar multiplication, tensor addition, element-wise (Hadamard) products, and contractions with matrices and vectors^13^. These operations are designed to preserve the TT structure but they generally lead to an increase in the TT ranks of the resulting tensor. A critical step is the so-called rounding procedure, denoted as ${{\textsf {rndg}}}(\,\cdot \,)$, which “recompresses” the tensor by computing a new TT representation with smaller ranks while controlling the approximation error^13^. Given a tensor $\mathcalligra{\scriptstyle A}\:$ in TT-format, the “rounded” tensor ${{\textsf {rndg}}}(\mathcalligra{\scriptstyle A}\:)$ satisfies the inequality $\Vert\mathcalligra{\scriptstyle A}\:- {{\textsf {rndg}}}(\mathcalligra{\scriptstyle A}\:)\Vert _{\mathcalligra{\scriptstyle F}}\: \le \tau \Vert \mathcalligra{\scriptstyle A}\:\Vert _{\mathcalligra{\scriptstyle F}}\:$, where $\tau$ is a threshold parameter that makes a trade-off between accuracy and computational costs possible. On the one hand, smaller values of $\tau$ can preserve a better accuracy but implies higher TT ranks, hence increasing the computational costs and storage requirements. On the other hand, larger values of $\tau$ lead to a better compression with lower ranks, thus reducing computational complexity at the expense of accuracy. This balance between accuracy and efficiency must be carefully calibrated according to the specific requirements of the application at hand.

Finally, it is worth mentioning the cross-approximation technique^60^, which allows us to compute the TT representation of a tensor by sampling only a small subset of its entries. This algorithm makes the calculation of a TT decomposition very efficient since in many applications, mainly when dealing with function-generated tensors, computing and storing the full tensor is prohibitively expensive or impossible. Cross-interpolation typically employs the MaxVol algorithm^62^, a sophisticated sampling strategy that selectively chooses optimal rows and columns from the tensor to construct its TT representation. In our context, cross-interpolation proves particularly valuable for efficiently representing the right-hand side term $f$, allowing us to work with high-dimensional data while maintaining computational tractability.

### ${\hbox {T}}^2{\hbox {S}}^2$ algorithm for convection-diffusion-reaction equations

We consider the problem defined by (1), (2), and (3) defined on the *d*-dimensional hypercube $\Omega \subset \mathbbm {R}^{d}$, with boundary conditions $\partial \Omega$. Therein, $\varepsilon$ is the (scalar) diffusion coefficient, ${\varvec \beta }=(\beta _{\ell })_{\ell =1,2,\ldots ,d}$ is the *d*-sized vector-valued convection field, $\rho$ is the scalar reaction coefficient, $b$ is the right-hand side source term. The coefficients $\varepsilon$, ${\varvec \beta }$, and $\rho$ can be constant or varying in space as a function of the multidimensional position vector ${\bf{x}}\in \mathbbm {R}^{d}$. In the last case, we assume that these coefficients satisfy appropriate regularity conditions so that problem (1)–(3) is well-posed. For example, we can assume that (*i*) $\varepsilon ({\bf{x}})$ is *uniformly positive*, i.e., there exists a strictly positive constant $\epsilon _0$ such that $\varepsilon ({\bf{x}})\ge \epsilon _0$ for all ${\bf{x}}\in \Omega$; (*ii*) the components of ${\varvec \beta }$ are smooth (e.g., at least $\beta _{\ell }\in C^{1}(\Omega )$) and bounded; (*iii*) $\rho$ is bounded and non-negative, i.e., $\rho \ge 0$. In such a case, we also assume that a sufficiently accurate, low-rank representation in TT format exists for all non-constant coefficients. If these coefficients are low-rank, we can obtain their TT representation through an application of the cross-interpolation algorithm.

The discrete diffusion operator. The low-rank, TT representation of $\varepsilon$ reads as $\varepsilon ^{TT}=\varepsilon _{1}\bowtie \varepsilon _{2}\bowtie \ldots \bowtie \varepsilon _{d}$, where the cores are given in the core notation by

$$\begin{aligned} \varepsilon _{\ell } = \left[ \begin{array}{cccc} \varepsilon _{\ell }(1,:,1) & \varepsilon _{\ell }(1,:,2) & \cdots & \varepsilon _{\ell }(1,:,r_{\ell }) \\[0.25em] \varepsilon _{\ell }(2,:,1) & \varepsilon _{\ell }(2,:,2) & \cdots & \varepsilon _{\ell }(2,:,r_{\ell }) \\[0.25em] \vdots & \vdots & \ddots & \vdots \\[0.25em] \varepsilon _{\ell }(r_{\ell -1},:,1) & \varepsilon _{\ell }(r_{\ell -1},:,2) & \cdots & \varepsilon _{\ell }(r_{\ell -1},:,r_{\ell )} \end{array} \right] , \,\, \ell =1,2,\ldots ,d. \end{aligned}$$

Then, for $\alpha _{\ell -1}=1,2,\ldots ,r_{\ell -1}$ and $\alpha _{\ell }=1,2,\ldots ,r_{\ell }$, we set $E_{\ell }(\alpha _{\ell -1},:,:,\alpha _{\ell })=\text {diag}\big (\varepsilon _{\ell }(\alpha _{\ell -1},:,\alpha _{\ell })\big )$, so that every $E_{\ell }(\alpha _{\ell -1},:,:,\alpha _{\ell })$ is a two-dimensional, diagonal matrix with $\varepsilon _{\ell }(\alpha _{\ell -1},:,\alpha _{\ell })$ along the diagonal. Using such a notation, we rewrite $\varepsilon ^{TT}$ as $\mathcalligra{\scriptstyle E}\:^{TT}= \mathcalligra{\scriptstyle E}\:_{1}\bowtie \mathcalligra{\scriptstyle E}\:_{2}\bowtie \ldots \bowtie \mathcalligra{\scriptstyle E}\:_{d}$, where each core of this TT representation is the block matrix

$$\begin{aligned} \mathcalligra{\scriptstyle E}\:_{\ell } = \left[ \begin{array}{cccc} E_{\ell }(1,:,:,1) & E_{\ell }(1,:,:,2) & \cdots & E_{\ell }(1,:,:,r_{\ell }) \\[0.25em] E_{\ell }(2,:,:,1) & E_{\ell }(2,:,:,2) & \cdots & E_{\ell }(2,:,:,r_{\ell }) \\[0.25em] \vdots & \vdots & \ddots & \vdots \\[0.25em] E_{\ell }(r_{\ell -1},:,:,1) & E_{\ell }(r_{\ell -1},:,:,2) & \cdots & E_{\ell }(r_{\ell -1},:,:,r_{\ell }) \end{array} \right] , \,\, \ell =1,2,\ldots ,d. \end{aligned}$$

For $\ell =1,2,\ldots ,d$, we let $I_{\ell }(\alpha _{\ell -1},:,:,\alpha _{\ell })\in \mathbbm {R}^{n_{\ell }\times n_{\ell }}$ be the $n_{\ell }\times n_{\ell }$-sized identity matrix for all $\alpha _{\ell -1}=1,2,\ldots ,r_{\ell -1}$ and $\alpha _{\ell }=1,2,\ldots ,r_{\ell }$. Finally, let $\Delta ^{n}_{\ell }$ be the one-dimensional discretization of the partial second derivative $\partial ^2/{\partial x_{\ell }}$ along the direction $\ell$. Precisely, $\Delta ^{n}_{\ell } = S_n\, D^2$ is the $\big ((n+1)\times (n+1)\big )$-sized matrix operator $D^2$ introduced in the left-hand side of Eq. (9), premultiplied for the matrix that shifts the evaluation to the collocation grid. We recall that $n$ is the polynomial degree of the spectral collocation approximation. The second order operator can be represented exactly as $\Delta = \mathcalligra{\scriptstyle D}\:= \mathcalligra{\scriptstyle D}\:_{1}\bowtie  \mathcalligra{\scriptstyle D}\:_{2}\bowtie \ldots \bowtie \mathcalligra{\scriptstyle D}\:_{d}$, where each $\mathcalligra{\scriptstyle D}\:_{\ell }$ contains $\Delta ^{n}_{\ell }$. The cores $\mathcalligra{\scriptstyle D}\:_{\ell }$ are given by:

$$\begin{aligned}  \mathcalligra{\scriptstyle D}\:_1 = \left[ \begin{array}{ccccc} \Delta ^{n}_{1}(1,:,:,1)&I_{1}(1,:,:,2)&\cdots&I_{1}(1,:,:,d-1)&I_{1}(1,:,:,d) \end{array} \right] , \end{aligned}$$

$$\begin{aligned}  \mathcalligra{\scriptstyle D}\:_{\ell } = \left[ \begin{array}{ccccccc} {} I_{\ell }(1,:,:,1) & & & & & & \\ {} & I_{\ell }(2,:,:,2) & & & & & \\ {} & & \ddots & & & & \\ {} & & & \Delta ^{n}_{\ell }(\ell ,:,:,\ell ) & & & \\ {} & & & & \ddots & & \\ {} & & & & & I_{\ell }(d-1,:,:,d-1) & \\ {} & & & & & & I_{\ell }(d,:,:,d) \end{array} \right] , \end{aligned}$$

$$\begin{aligned} \mathcalligra{\scriptstyle D}\:_{d} = \left[ \begin{array}{c} I_{d}(1,:,:,1) \\ I_{d}(2,:,:,1) \\ \vdots \\ I_{d}(d-1,:,:,1)\\ \Delta ^{n}_{d}(d,:,:,1) \end{array} \right] . \end{aligned}$$

Finally, we consider the approximation $\varepsilon \Delta \approx \mathscr {E}\cdot  \mathcalligra{\scriptstyle D}\:$, where the dot product is defined at algorithm 4 in^13^, and the superscript $TT$ is dropped.

The discrete convection operator.

A similar reasoning can be applied to the first-order differential operator ${\varvec \beta }\cdot \nabla$, where ${\varvec \beta }$ is a vector coefficient with $d$ components, i.e., ${\varvec \beta }=\big (\beta _{1},\beta _{2},\ldots ,\beta _{d}\big )^T$, and the partial differential operator $\nabla$ has also $d$ components, i.e., $\nabla =(\partial _1,\partial _2,\dots ,\partial _{d})^T$ with the abbreviation $\partial _{k}=\partial /{\partial x_{k}}$, for $k=1,2,\ldots ,d$. We construct a TT representation of ${\varvec \beta }\cdot \nabla$ by splitting the directional derivative along ${\varvec \beta }$ into its directional components and then introducing the TT representation of each term:

$$\begin{aligned} {\varvec \beta }\cdot \nabla = \sum _{k=1}^{d}\beta _{k}\partial _{k} \approx \sum _{k=1}^{d}\beta ^{TT}_{k}\partial ^{TT}_{k} = {\varvec \beta }^{TT}\cdot \nabla ^{TT}, \end{aligned}$$

or, equivalently, by saying that $({\varvec \beta }\cdot \nabla )^{TT}={\varvec \beta }^{TT}\cdot \nabla ^{TT}$, where ${\varvec \beta }^{TT}=\big (\beta ^{TT}_{1},\beta ^{TT}_{2},\ldots ,\beta ^{TT}_{d}\big )$ and $\nabla ^{TT}=(\partial ^{TT}_1,\partial ^{TT}_2,\dots ,\partial ^{TT}_{d})$. The TT decomposition of the *k* component $\beta ^{TT}_{k}=\beta ^{(k)}_{1}\bowtie \beta ^{(k)}_{2}\bowtie \ldots \bowtie \beta ^{(k)}_{d}$ is written using the cores $\beta ^{(k)}_{\ell }$. As for the diffusion operator, we set $B_{\ell }^{(k)}(\alpha _{\ell -1},:,:,\alpha _\ell ) = \textrm{diag}\left( \beta _{\ell }^{(k)}(\alpha _{\ell -1},:,\alpha _\ell ) \right)$; we rewrite $\beta _{k}^{TT}$ as $\mathcalligra{\scriptstyle B}\:_{k}^{TT}=  \mathcalligra{\scriptstyle B}\:_{1}^{(k)}\bowtie  \mathcalligra{\scriptstyle B}\:_{2}^{(k)}\bowtie \ldots \bowtie \mathcalligra{\scriptstyle B}\:_{d}^{(k)}$ where:

$$\begin{aligned} \mathcalligra{\scriptstyle B}\:^{(k)}_{\ell } = \left[ \begin{array}{cccc} B_{\ell }^{(k)}(1,:,:,1) & B_{\ell }^{(k)}(1,:,:,2) & \cdots & B_{\ell }^{(k)}(1,:,:,r_{\ell }^{(k)}) \\[0.5em] B_{\ell }^{(k)}(2,:,:,1) & B_{\ell }^{(k)}(2,:,:,2) & \cdots & B_{\ell }^{(k)}(2,:,:,r_{\ell }^{(k)}) \\[0.5em] \vdots & \vdots & \ddots & \vdots \\[0.5em] B_{\ell }^{(k)}(r_{\ell -1}^{(k)},:,:,1) & B_{\ell }^{(k)}(r_{\ell -1}^{(k)},:,:,2) & \cdots & B_{\ell }^{(k)}(r_{\ell -1}^{(k)},:,:,r_{\ell }^{(k)}) \end{array} \right] . \end{aligned}$$

The TT representation of the *k*-th directional derivative $\partial ^{TT}_{k}=\mathcalligra{\scriptstyle N}\:_{1}^{(k)}\bowtie \mathcalligra{\scriptstyle N}\:_{2}^{(k)}\bowtie \ldots \bowtie \mathcalligra{\scriptstyle N}\:_{d}^{(k)}$ follows the same principals as the second order operator. We denote by $\partial _{k}^{n}$ the (matrix) operator representing the one-dimensional partial derivative along the direction *k*, premultiplied by the interpolation matrix $S_n$. The resulting cores $\mathcalligra{\scriptstyle N}\:_{\ell }^{(k)}$ for all *k* are given by

$$\begin{aligned}\mathcalligra{\scriptstyle N}\:_{1}^{(k)} = \left[ \begin{array}{ccccc} \partial _{1}^{n}(1,:,:,1)&I_{1}(1,:,:,2)&\cdots&I_{1}(1,:,:,d-1)&I_{1}(1,:,:,d) \end{array} \right] , \end{aligned}$$

$$\begin{aligned} \mathcalligra{\scriptstyle N}\:_{\ell }^{(k)} = \left[ \begin{array}{cccccc} {} I_{\ell }(1,:,:,1) & & & & & \\ {} & I_{\ell }(2,:,:,2) & & & & \\ {} & & \ddots & & & \\ {} & & & \partial _{k}^{n}(\ell ,:,:,\ell ) & & \\ {} & & & & \ddots & \\ {} & & & & I_{\ell }(d-1,:,:,d-1) & \\ {} & & & & & I_{\ell }(d,:,:,d) \end{array} \right] ,\,\, \ell =2,\ldots ,d-1, \end{aligned}$$

$$\begin{aligned} \mathcalligra{\scriptstyle N}\:_{d}^{(k)} = \left[ \begin{array}{c} I_{d}(1,:,:,1) \\ I_{d}(2,:,:,1) \\ \vdots \\ I_{d}(d-1,:,:,1) \\ \partial _{d}^{n}(d,:,:,1) \end{array} \right] . \end{aligned}$$

With this notation, the advection operator can be written as

$$\begin{aligned} {\varvec \beta }\cdot \nabla \approx \sum _{k=1}^{d}\mathcalligra{\scriptstyle B}\:^{TT}_{k}\cdot \partial ^{TT}_{k} = \sum _{k=1}^{d}\mathcalligra{\scriptstyle D}\:_{k}\cdot \partial _{k} \end{aligned}$$

where the $\cdot$ on the right hand side is the matrix-by-matrix product defined at algorithm 4 in^13^, and the superscript $TT$ is dropped.

The discrete reaction operator. Following the same approach used for the diffusion and convection operators, we now construct the discrete reaction operator in TT format. The low-rank, TT representation of $\rho$ reads as $\rho ^{TT}=\rho _{1}\bowtie \rho _{2}\bowtie \ldots \bowtie \rho _{d}$, where the cores are given in the core notation by

$$\begin{aligned} \rho _{\ell } = \left[ \begin{array}{cccc} \rho _{\ell }(1,:,1) & \rho _{\ell }(1,:,2) & \cdots & \rho _{\ell }(1,:,r_{\ell }) \\[0.25em] \rho _{\ell }(2,:,1) & \rho _{\ell }(2,:,2) & \cdots & \rho _{\ell }(2,:,r_{\ell }) \\[0.25em] \vdots & \vdots & \ddots & \vdots \\[0.25em] \rho _{\ell }(r_{\ell -1},:,1) & \rho _{\ell }(r_{\ell -1},:,2) & \cdots & \rho _{\ell }(r_{\ell -1},:,r_{\ell )} \end{array} \right] , \,\, \ell =1,2,\ldots ,d. \end{aligned}$$

Then, we introduce the reaction term $\mathcalligra{\scriptstyle R}\:$, which is represented through the core sequence $\mathcalligra{\scriptstyle R}\:^{TT}=\mathcalligra{\scriptstyle R}\:_{1}\bowtie \mathcalligra{\scriptstyle R}\:_{2}\bowtie \ldots \bowtie \mathcalligra{\scriptstyle R}\:_{d}$, where each core corresponds to a spatial dimension. The $\ell$-th core is given by

$$\begin{aligned} \mathcalligra{\scriptstyle R}\:_{\ell } = \left[ \begin{array}{cccc} R_{\ell }(1,:,:,1) & R_{\ell }(1,:,:,2) & \cdots & R_{\ell }(1,:,:,r_{\ell }) \\[0.25em] R_{\ell }(2,:,:,1) & R_{\ell }(2,:,:,2) & \cdots & R_{\ell }(2,:,:,r_{\ell }) \\[0.25em] \vdots & \vdots & \ddots & \vdots \\[0.25em] R_{\ell }(r_{\ell -1},:,:,1) & R_{\ell }(r_{\ell -1},:,:,2) & \cdots & R_{\ell }(r_{\ell -1},:,:,r_{\ell }) \end{array} \right] , \,\, \ell =1,2,\ldots ,d, \end{aligned}$$

where, for $\alpha _{\ell -1}=1,2,\ldots ,r_{\ell -1}$ and $\alpha _{\ell }=1,2,\ldots ,r_{\ell }$, we set $R_{\ell }(\alpha _{\ell -1},:,:,\alpha _{\ell })=\text {diag}(\rho _{\ell }(\alpha _{\ell -1},:,\alpha _{\ell })$, so that every $R_{\ell }(\alpha _{\ell -1},:,:,\alpha _{\ell })$ is a two-dimensional, diagonal matrix with $\rho _{\ell }(\alpha _{\ell -1},:,\alpha _{\ell })$ along the diagonal. Finally, we note that the diagonal structure of the matrices reflects the multiplicative nature of the reaction term.

The space–time formulation.

The final space–time differential operator includes the time derivative term:

$$\begin{aligned} L_{n}:=D_{t}\otimes \, \bigotimes _{\ell =1}^{d}I_{\ell } + I_{t}\otimes \bigg [ -\mathcalligra{\scriptstyle E}\cdot\mathcalligra{\scriptstyle D}+ \sum _{k=1}^{d} \mathcalligra{\scriptstyle B}\: _{k}\cdot  \mathcalligra{\scriptstyle N} _{k} +  \mathcalligra{\scriptstyle R}\: \bigg ], \end{aligned}$$

where $D_{t}$ e $I_{t}$ are the discrete derivative and the identity matrix relative to the time discretization, where the time operator is the approximation of $\partial /{\partial t}$. $\otimes$ is the tensor product of two arrays, and $\bigotimes$ is the tensor product of multiple arrays. $\mathcalligra{\scriptstyle E}\cdot \mathscr {D}$ is the TT approximation of the discrete diffusion operator $\varepsilon \Delta$. $\sum _{k=1}^{d} \mathcalligra{\scriptstyle B}_{k}\cdot  \mathcalligra{\scriptstyle N}_{k}$ is the TT approximation of the discrete advection operator ${\varvec \beta }\cdot \nabla$, and $\mathcalligra{\scriptstyle R}$ is the TT approximation of the discrete reaction operator. The superconsistent version of the scheme is achieved by evaluating the terms $\mathcalligra{\scriptstyle B}$, $\mathcalligra{\scriptstyle E}$ and $\mathcalligra{\scriptstyle R}$ on the superconsistent grid.

**Fig. 12:**
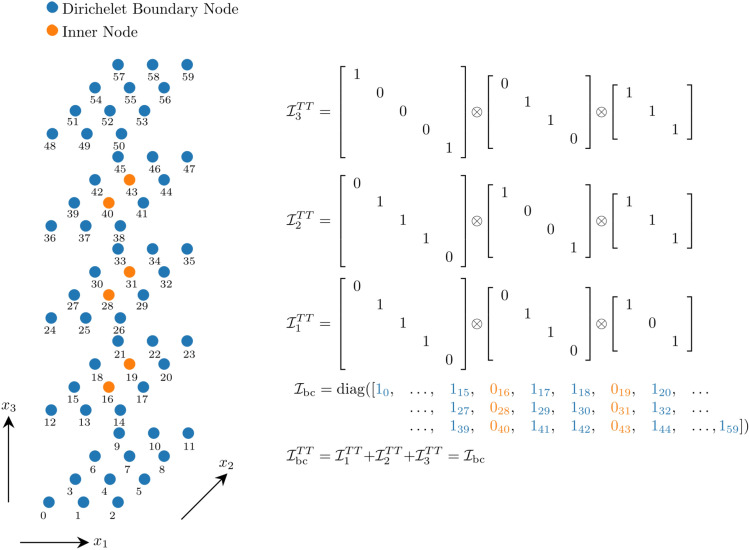
In this picture, a three dimensional example illustrates the procedure to represent the diagonal matrix with ones corresponding to the boundary nodes and zeros elsewhere. The matrix $\mathcal {I}^{TT}_{\textrm{bc}}$ is added to the overall system matrix with zero rows corresponding to the boundary nodes.

Boundary conditions. To apply Dirichelet boundary conditions in full format we set to zero the rows that correspond to the boundary points. In TT format that is achieved by setting to zero the first and the last rows of the one dimensional discrete operators described in the previous paragraphs. In full format we need to add a diagonal matrix that is one corresponding to the boundary nodes and zero elsewhere, namely $\mathcal {I}_{\textrm{bc}}$, in Fig. 12. Figure 12 presents a simple three dimensional grid with highlighted the boundary and the inner nodes. $\mathcal {I}_{i}^{TT}$ sets to one the elements corresponding to the $x_i$ direction. $\mathcal {I}^{TT}_{\textrm{bc}}$ is the sum of $\mathcal {I}_{i}^{TT}$ with $i=1,2,3$, and exactly corresponds to $\mathcal {I}_{\textrm{bc}}$.

## Supplementary Information

Supplementary Information.

## Data Availability

Data is provided within the manuscript or supplementary information files.
